# Recombination Events Shape the Genomic Evolution of Infectious Bronchitis Virus in Europe

**DOI:** 10.3390/v13040535

**Published:** 2021-03-24

**Authors:** Krisztina Bali, Ádám Bálint, Attila Farsang, Szilvia Marton, Borbála Nagy, Eszter Kaszab, Sándor Belák, Vilmos Palya, Krisztián Bányai

**Affiliations:** 1 Institute for Veterinary Medical Research, Centre for Agricultural Research, 1143 Budapest, Hungary; krisz.bali@gmail.com (K.B.); martonsil@gmail.com (S.M.); nagyborka@hotmail.com (B.N.); eszter.kaszab@gmail.com (E.K.); 2 Veterinary Diagnostic Directorate, National Food Chain Safety Office, 1143 Budapest, Hungary; BalintAd@nebih.gov.hu; 3 Ceva-Phylaxia, Ceva Sante Animale, 1107 Budapest, Hungary; attila.farsang@ceva.com (A.F.); vilmos.palya@ceva.com (V.P.); 4 Department of Biomedical Sciences and Veterinary Public Health (BVF), Swedish University of Agricultural Sciences (SLU), 750 07 Uppsala, Sweden; sandor.belak@slu.se

**Keywords:** infectious bronchitis, genomic epidemiology, genomic evolution

## Abstract

Infectious bronchitis of chicken is a high morbidity and mortality viral disease affecting the poultry industry worldwide; therefore, a better understanding of this pathogen is of utmost importance. The primary aim of this study was to obtain a deeper insight into the genomic diversity of field infectious bronchitis virus (IBV) strains using phylogenetic and recombination analysis. We sequenced the genome of 20 randomly selected strains from seven European countries. After sequencing, we created a genome sequence data set that contained 36 European origin field isolates and 33 vaccine strains. When analyzing these 69 IBV genome sequences, we identified 215 recombination events highlighting that some strains had multiple recombination breaking points. Recombination hot spots were identified mostly in the regions coding for non-structural proteins, and multiple recombination hot spots were identified in the nsp2, nsp3, nsp8, and nsp12 coding regions. Recombination occurred among different IBV genotypes and involved both field and vaccine IBV strains. Ninety percent of field strains and nearly half of vaccine strains showed evidence of recombination. Despite the low number and the scattered geographical and temporal origin of whole-genome sequence data collected from European Gammacoronaviruses, this study underlines the importance of recombination as a major evolutionary mechanism of IBVs.

## 1. Introduction

Infectious bronchitis (IB) of chicken (Gallus gallus domesticus) is a leading cause of morbidity and mortality affecting the poultry industry worldwide [1]. The etiological agent, infectious bronchitis virus (IBV), belongs to the genus *Gammacoronavirus*, family *Coronaviridae* [2]. IBV can be classified based on pathogenic, genetic, and immunologic features into pathotypes, genotypes, and serotypes, respectively [3,4,5,6]. The spectrum of tissue tropism greatly influences disease manifestations; accordingly, different strains may be associated with respiratory syndrome, interstitial nephritis, proventriculitis, and/or reproductive disease [1]. Serotyping was once an important tool to characterize IBV strains; however, it has become difficult to keep up with the growing number of antigenic variants having emerged in the field over the past few decades. Genetic sequencing of the spike protein coding gene is a reliable substitute of serology based characterization methods; in addition, it is widely available and readily adaptable in many laboratories [7,8]. Attenuated and inactivated vaccines against IB are in commercial use [9]. The development of IBV vaccines that provide wide heterotypic protection against multiple serotypes is challenging. Yet, some strains are more suitable to induce both humoral and cellular immunity than others, and thus, they are good candidates to maximize heterotypic protection against a wide range of wild-type strains.

The enveloped virion of IBV is 120 nm in diameter and encapsidates the 27 kilobase positive sense, single-stranded RNA genome. The genome contains 13 open reading frames, and it encodes four structural proteins and at least 18 mature non-structural proteins [1,2]. The viral genome mutates rapidly; the accumulation of point mutations is associated with a yearly evolutionary rate of up to 1.2 × 10^−3^ substitutions per nucleotide site [10,11]. The genetic drift that continuously produces novel antigenic variants is often accompanied by preceding or subsequent recombination events. The recombination of genomic RNA may lead to a rapid antigenic shift if key structural elements (i.e., spike protein coding gene) are involved. Evidence of recombination events that affect the spike protein coding region or other genomic regions has been published in the literature [12,13]. 

The recently proposed classification scheme of IBVs that is based on the S1 gene has classified IBVs into six genotypes (GI to GVI) and multiple lineages within one genotype (GI-1 to GI-27) [13]. Within the largest and most diverse IBV genotype, GI, four lineages (GI-1, GI-13, GI-16, and GI-19) are distributed in many parts of the world and represent the dominating genotypes on some continents. Some GI lineages are known to be restricted to a particular continent. For example, genotype GI-7, GI-15, GI-18, GI-22, and GI-24 strains have been described in parts of Asia, whereas GI-9 and GI-27 are widespread in North America. Genotype GI-21 has been identified as a unique strain in Europe, whereas genotypes GI-12 and GI-14 are shared between Europe and Africa [13]. The S1 gene fragment based molecular identification of IBV strains supports molecular epidemiological studies; however, this genomic region comprises roughly six percent of the entire genome. Because IBV is prone to recombination, template switching mechanisms involving genomic regions other than the S gene will be overseen in studies focusing exclusively on the S gene. Adaptive immune response that provides good heterotypic protection involves both humoral and cellular arms; therefore, understanding the diversity and the mechanisms of diversification along the entire genome could be paramount for designing more effective control measures. 

Despite the wide availability of sequencing technologies suitable for the easy generation of whole-genome sequences, a surprisingly low number of whole-genome sequences can be found for IBVs in public databases. To obtain a deeper insight into the genomic diversity of field IBV strains, we analyzed genome sequences originating from European countries. In addition to the sequences collected for 16 European origin wild-type strains plus 33 vaccine strains, we analyzed the genomic sequences of another 20 strains detected between 2010 and 2019 in five European countries. We determined the preferred sites of recombination in the genome structures and investigated the frequency of recombination among field and vaccine strains. 

## 2. Materials and Methods

### 2.1. Virus Isolates

The viruses (*n* = 20) used in this study represent a collection of IBV isolates from Europe detected over a 10 year period. Various partner companies, located in different countries, kindly provided the samples to CEVA-Phylaxia in Budapest. Additional isolates were obtained from the collection of the Hungarian National Food Chain Safety Office. All isolates originated from broiler-type chick hybrids (aged 7 to 40 days) with various clinical backgrounds. Trachea, kidney, and caecal tonsils were the most common organ types from which virus isolation was performed. See additional details in Table 1. Whenever data were available, the IBV vaccine type used in the affected flock is also listed. 

Tissue homogenates in PBS (1:10 dilution) were prepared from the organs and passed through a 0.22 μm filter. Then, two-hundred microliters of the homogenate were inoculated in the allantoic cavities of embryonated chicken eggs and incubated for 6–7 days at 37 °C. A sample was considered positive for IBV if the embryos in the inoculated eggs showed typical lesions (stunting and curling of the embryos), and RT-PCR [14] identified genomic RNA.

### 2.2. Nucleic Acid Extraction and Random Primed RT-PCR

To enrich viral genomic RNA from the harvested allantoic fluid, samples were centrifuged at 10,000× *g* for 5 min and passed through a 0.45 µm sterile syringe filter (Nantong FilterBio Membrane Co., Ltd., Nantong, Jiangsu, China). To 100 µL of the supernatant, thirty-eight microliters of reagent mixture containing Turbo DNase buffer, Turbo DNase (Ambion/Thermo Scientific, Waltham, MA, USA, 2 U/μL), RNase I (Thermo Scientific, Waltham, MA, USA, 10 U/μL), Benzonase (Novagen, Merck KGaA, Darmstadt, Germany, 25 U/μL), and nuclease-free water were added. The samples were incubated at 37 °C for 2 h, and then, the reaction was stopped at 75 °C for 10 min. Viral nucleic acid was extracted from the supernatant using the innuPREP Virus DNA/RNA Kit (Analytik Jena, Jena, Germany) according to the manufacturer’s instruction. 

For sequence-independent amplification of the extracted RNA, random primed RT-PCR was performed. First, the sample was denatured at 95 °C for 5 min with the primer FR26RV-N (5′-GCC GGA GCT CTG CAG ATA TCN NNN NN-3′) [15]. The reverse transcription was performed using 10 U AMV Reverse Transcriptase (Promega, Madison, WI, USA) starting at 25 °C for 10 min, followed by incubation at 42 °C for an hour, and then, terminated by an enzyme deactivation step at 70 °C for 15 min. 

The cDNA was used for PCR using 2.5 U Dream Taq DNA polymerase (Thermo Scientific, Waltham, MA, USA) in the presence of a single amplification primer, FR20RV (5′- GCC GGA GCT CTG CAG ATA TC-3′). The following heat profile was employed during amplification: the initial denaturation step at 95 °C for 3 min was followed by 40 cycles of 95 °C for 30 s, 48 °C for 30 s, and 72 °C for 2 min, and the final extension step was carried out at 72 °C for 5 min. The random amplified DNA was run on 1% agarose gel and stained with GelRed (Biotium). The obtained smear was excised and extracted from the gel using the Gel/PCR DNA Fragments Extraction kit (Geneaid Biotech Ltd., Taipei, Taiwan).

The concentration of purified DNA was measured with Qubit 2.0 equipment using the Qubit dsDNA BR Assay Kit (Thermo Scientific, Waltham, MA, USA).

### 2.3. Next-Generation Sequencing

Nucleotide sequences were determined by next-generation sequencing on an Ion Torrent PGM (Life Technologies/Thermo Fisher Scientific, Waltham, MA, USA) platform and/or on an Illumina^®^ NextSeq 500 sequencer (Illumina, San Diego, CA, USA) following the protocols described previously [16,17]. 

As for semiconductor sequencing (SCS), ten to 100 ng of random PCR products were fragmented enzymatically (NEBNext Fast DNA Fragmentation & Library Prep Set for Ion Torrent kit; New England Biolabs, Hitchin, United Kingdom). In brief, eight microliters of DNA were mixed with 1 µL of NEBNext DNA Fragmentation Reaction buffer, 0.5 µL MgCl2 (using a 10 mM stock), and 0.75 µL NEBNext DNA Fragmentation Master Mix. The mixture was incubated at 25 °C for 20 min, then at 70 °C for 10 min. The adaptor ligation was performed using reagents from the same kit, whereas barcoded adaptors were retrieved from the Ion Xpress Barcode Adapters (Thermo Fischer Scientific, Waltham, MA, USA). Reaction components were as follows: 2 µL T4 DNA Ligase Buffer for Ion Torrent, 2 µL barcode adapter mixture, 0.5 µL Bst DNA Polymerase, and 2 µL T4 DNA Ligase were combined with the fragmentation reaction mixture and nuclease-free water in a final volume of 20 µL. The mixture was incubated at 25 °C for 15 min and terminated at 65 °C for 5 min. After cooling on ice, two-point-five microliters 2 of Stop Buffer were added to the mixture. The barcoded library DNA samples were purified using the Gel/PCR DNA fragments extraction kit (Geneaid Biotech, Ltd., Taipei, Taiwan). Size selection was performed by agarose gel electrophoresis using 2% E-Gel SizeSelect II Agarose (Invitrogen, Carlsbad, CA, USA). Products between 300 and 350 bp were directly used in the PCR mixture of the NEBNext Fast DNA Fragmentation & Library Prep Set for Ion Torrent kit as recommended by the manufacturer (New England BioLabs, Hitchin, United Kingdom). The library DNA was purified on a spin-column and quantified. Subsequently, the library DNA was diluted to 10 pM, then clonally amplified by emulsion PCR. This step was carried out according to the manufacturer’s instructions using the Ion PGM Hi-Q View OT2 Kit on an Ion OneTouch 2 instrument. Templated beads were enriched on an Ion OneTouch ES machine, and the 200 bp sequencing protocol was performed on a 316 chip using the Ion Torrent PGM (Life Technologies/Thermo Fisher Scientific, Waltham, MA, USA) semiconductor sequencing equipment.

Concerning the reversible terminator sequencing (RTS) method, the Illumina^®^ Nextera XT DNA Library Preparation Kit (Illumina, San Diego, CA, USA) and the Nextera XT Index Kit v2 Set A (Illumina, San Diego, CA, USA) were used to prepare Illumina specific libraries. DNA samples were diluted to 0.2 ng/μL in nuclease-free water (Promega, Madison, WI, USA) in a final volume of 2.5 μL. Reaction components were used at a reduced volume. For the tagmentation reaction, five microliters of Tagment DNA buffer with 2.5 μL of Amplicon Tagment Mix were used. During tagmentation, the samples were incubated at 55 °C for 6 min, using the GeneAmp PCR System 9700 (Applied Biosystems/Thermo Fisher Scientific, Foster City, CA, USA). The samples were then allowed to cool to 10 °C before the addition of 2.5 μL of the Neutralize Tagment buffer. Neutralization was performed for 5 min at room temperature. A total of 7.5 μL of the Nextera PCR Master Mix and 2.5 μL each of the i5 and i7 index primers were added to the tagmented DNA samples. The index primers were incorporated into library DNA via 12 PCR cycles (each cycle consisted of the following steps: 95 °C for 10 s, 55 °C for 30 s, followed by 72 °C for 30 s). Following the PCR cycles, the samples were held at 72 °C for 5 min and then at 10 °C. Libraries were purified using the Gel/PCR DNA Fragments Extraction Kit (Geneaid Biotech Ltd., Taipei, Taiwan). The concentration of the purified libraries was measured, then the libraries were pooled and denatured. The denatured library pool at a final concentration of 1.5 pM was loaded onto a NextSeq 500/550 Mid Output flow cell and sequenced using an Illumina^®^ NextSeq 500 sequencer (Illumina, San Diego, CA, USA).

### 2.4. Sequence Data and Analysis Tools 

Sequence reads generated by the Illumina and Ion Torrent platforms were assembled and analyzed by Geneious Prime (Biomatters Ltd., New Zealand) (GenBank Accession Nos. MT984583-MT984602). Reference genomic sequences of IBV strains were downloaded for comparison (including genome assembly and downstream applications) from the NCBI nucleotide sequence database (http://www.ncbi.nlm.nih.gov, accessed on 1 January 2021) (Table 2). 

The classification of strains based on the S1 gene was according to Valastro and co-workers [13]. Multiple alignments were prepared using the codon based Clustal W program. Phylogenetic trees were constructed using the maximum likelihood method and general time reversible model and the neighbor-joining method and *p*-distance model in MEGA X [18]. 

The IBV complete genome sequences were screened for recombination breakpoints using Recombination Detection Program 4 (RDP4, Version 4.97) [19]. Sequences involved in the recombination analyses originated from our collection (*n* = 20) or were obtained from GenBank (*n* = 49). The seven methods used for the analysis were RDP [20], GENECONV [21], BootScan [22], maximum chi squared (MaxChi) [23], Chimaera [24], SiScan [25], and 3Seq [26]. Default settings were used for most algorithms. To obtain a conservative estimate, a recombination event was accepted only when detected by five or more methods implemented in the program, with a *p*-value less than 5 × 10^-4^. The recombination events were further analyzed by using the SimPlot software (Version 3.5.1) [27]. To illustrate the complex pattern of recombination events, we generated Sankey diagrams using the Power-user add-in smart tool for Excel. In brief, the results obtained by the recombination analysis were summarized in a matrix, where we assigned values to minor or major parental strains that we finally visualized in individual Sankey charts; each chart displays the analyses related to a particular genotype identified during the study. 

## 3. Results and Discussion

Whole-genome sequence data were generated by the SCS method on an Ion Torrent PGM sequencer and/or by the RTS method on an Illumina Nextseq 500 platform. Following the technology shift, we applied the Illumina sequencer, which resulted in considerably greater per sample sequence read outputs at reduced per base costs. In this study, the typical sequence read outputs per sample for SCS and RTS in barcoded multiplex runs were ~70 thousand (range, 27.5 to 136.9 thousand) and ~4 million (range, 3.4 to 4.3 Million), respectively. The average sequencing depth for SCS and RTS was ~400× (range, 160× to 929×) and ~11,000× (range, 5298× to 14,453×), respectively. On average, seventy-nine percent (range, 27.4% to 94.2%) of the generated sequence reads mapped onto the assembled consensus genomes, suggesting that the enrichment protocol we adapted for IBV from allantoic fluid met our expectations. The SCS method alone or in combination with the RTS method permitted the consensus genome of all 20 strains to be determined.

The consensus genomes of IBV strains started with the sequence ACUUAAG and ended with the poly-A tract [2]. The whole-genome lengths without the poly-A tract varied between 27,348 and 27,845 nucleotides (nt). In terms of sequence length, variable genomic regions included ORF1ab (range, 19,827 to 19,896 nt in length), ORF1b (7959–7971 nt), spike protein gene (3489–3510 nt), ORF 3a (174 nt) ORF3b (177–195 nt), envelope and membrane protein genes (279–330 nt and 669–684 nt, respectively), ORF4b (285–288 nt), ORF4c (162–171 nt), and ORF6b (222–225 nt). ORF5a (198 nt), ORF5b (249 nt), and the nucleoprotein coding gene (1230 nt) did not show sequence length variation. 

**Figure 1:**
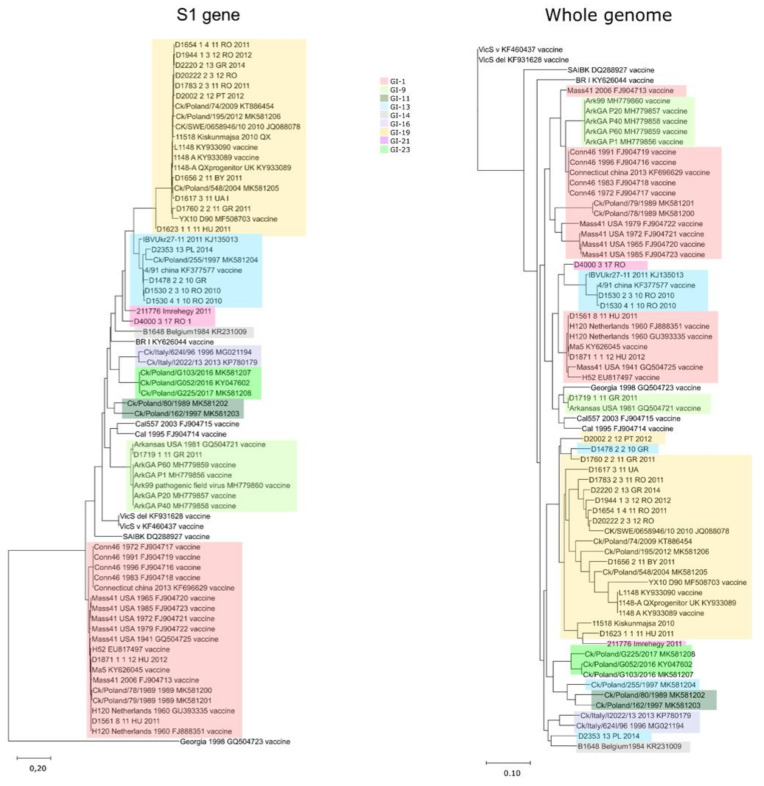
Phylogenetic trees generated from the S1 gene (on the left) and whole-genome (on the right) sequences. The S1 gene based genotypes are color-coded (see the panel in the middle).

Genetic classification based on the spike protein coding region [13] identified five genotypes among the 20 study strains, including two GI-1 strains from Hungary, one GI-9 (Arkansas-like) strain from Greece, four GI-13 (4/91-like) from Greece, Romania, and Poland, 11 GI-19 (QX-like) from six countries, and two GI-21 strains, one from Hungary and one from Romania (Figure 1). Whole-genome based sequence similarities among these 20 European strains ranged between 87% and near 100%. After the inclusion of GenBank sequence records, among the 36 European field strains, the range of intragenotype whole-genome sequence similarity was found to fall between 89.9% (genotype GI-13) and 99.9% (e.g., genotype GI-1). Whole-genome based intergenotype similarity values were, in general, lower (range, 87.2 to 95.6%), but overlapped to some extent with intragenotype similarity values. In a few cases, field strains shared great genome-wide similarity (≥99.5%) with vaccine strains (e.g., D1530 and 4/91, D1561 and Ma5, and D1719 and Arkansas). This finding was consistent with the isolation of a vaccine strain, or a vaccine-derived revertant strain that could have persisted in the flock for some weeks following the administration of vaccine [28,29,30]. Phylogenetic analysis of the S1 gene and the whole-genome based alignments revealed a distinct pattern of branching (Figure 1, Supplementary File S1). The S1 based tree was, in general, in accordance with the genotype assignment [13], whereas the whole-genome based tree followed a more complex pattern. In this analysis, we observed that a Hungarian GI-21 strain clustered with the Hungarian GI-19 strain in the whole-genome based phylogenetic analysis. Similarly, a GI-13 strain from Greece clustered with the GI-19 strain in the whole-genome based tree. The genotype GI-1 field and vaccine strains shared whole-genome sequence based similarity with genotype GI-11, GI-21, and GI-23 strains, respectively. Furthermore, a genotype IV vaccine strain clustered with some genotype GI-9 vaccine and vaccine-derived field strains in the whole-genome based tree, suggesting that inter-genotype recombination may occur [31,32,33]. Strains were found to cluster with other strains in a genotype-independent manner, as seen in phylogenetic analysis of ORFs other than the S1 protein coding gene. This finding corroborated the difference seen when S1 based and whole-genome based trees were compared (see Supplementary File S1). In general, only clonal variants of some vaccine strains (e.g., Massachusetts- and Connecticut-type strains), a few field strains originating from shared geographic location and some field isolates closely related to a particular vaccine strain, tended to form shared cluster in all phylogenetic trees. The relative positions of these clusters with other clusters or unique sequences, however, displayed very diverse branching patterns. 

**Figure 2:**
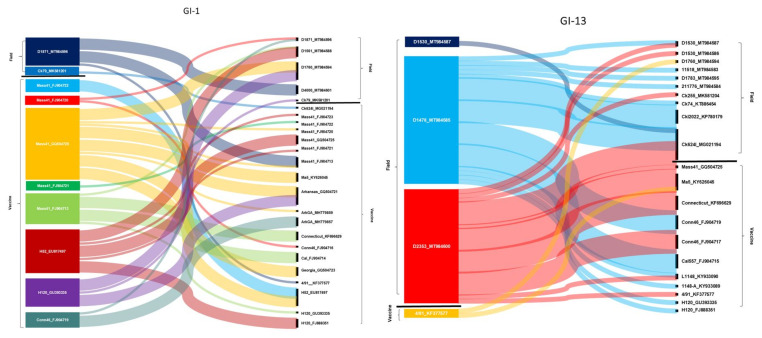
A summary of recombination events seen in field and vaccine origin IBV strains. Recombinant strains are listed on the left, the (putative) parental strains on the right. The relative thickness of the ribbons connecting strains on the left with strains on the right defines the (putative) major and minor parental strains (major, thick; minor, thin), although this relation could not be predicted for all cases. For simplicity, we show only two representative Sankey charts (generated for genotypes GI-1 and GI-13); the remainders are shared in the Supplementary File S2.

Based on findings of phylogenetic analyses and data from the literature that indicated that recombination events are common among IBV strains and involve regions across the entire genome [34], we further analyzed the frequency and location of possible recombination sites using whole-genome sequence data. We took into account only those events that were predicted by at least five different recombination searching algorithms. Putative major and minor parental genomes of the recombinants were categorized as the outputs that the analysis tools indicated. With these test parameters, a total of 215 recombination events within 51 strains (including 18 study strains, 13 field strains from GenBank, and 20 vaccine strains from GenBank) were identified (Figure 2). Some strains had multiple recombination breaking points, a finding that is congruent with the results previously reported from Asia. For example, Ren and coworkers [35] found that a Chinese origin field isolate emerged from multiple recombination events with at least three recombination breakpoints between a field isolate (designated as LJL/110302-like) and two vaccine strains (Conn-like and 4/91-like). Additionally, Zhou and coworkers described a recombinant field strain isolated from a vaccinated flock whose genome was found to have originated from multiple template switches among field strains (Ck/CH/LSC/99I-, tI/CH/LTD3/03-type) and vaccine strains (QX-like, 4/91-type) [36]. In our analyses performed on European origin field strains, the majority of recombination events were detected among field strains and between field and vaccine strains. Vaccine strains themselves were found to recombine relatively rarely. However, it is worth mentioning that the examined vaccine strains were older (time span for isolation of parental strains, 1941 to 2017) than the field strains of this study (time span for study strains and for GenBank sequences was 2010 to 2019 and 1984 to 2017, respectively). Therefore, to adequately interpret the data, it needs to be highlighted that divergence between old strains and recently circulating strains can be substantial, and some ancestral clones and lineages that were used to develop vaccine could be already extinct.

**Figure 3:**
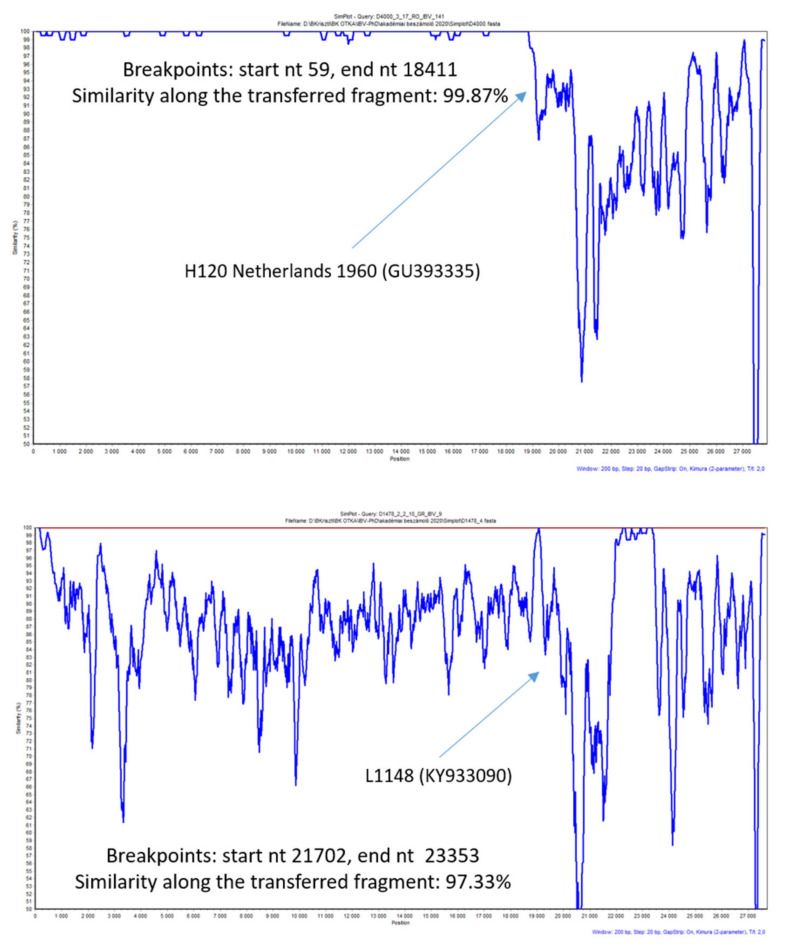
Simplot analysis together with sequence similarity data calculated for the transferred genomic region may help predict whether a putative recombination event occurred more recently or in the past. For putative vaccine-field recombinant IBVs, the similarity values over 99% might be indicative of a more recent recombination event between a particular vaccine strain and a field strain.

Each time when a vaccine strain was implicated in a predicted genomic RNA transfer event, Simplot analysis was executed to see the genome-wide similarity along a sliding window (Figure 3). This analysis identified at least 10 events when a transferred genomic fragment retained over 99% similarity with the putative vaccine strain and 35 events when the similarity with a putative vaccine origin sequence fell below 99%. The similarity values over 99% were thought to represent more recent recombination events between vaccine and field strains with a greater likelihood that the event just preceded or was co-incidental with the actual isolation of the particular field strain. Similar conclusions were made in previous reports when analyzing some North American and Asian origin field isolates of IBV [34,36]. 

The information about shared genotype specificity between vaccine and isolated strains (Table 1) supported the hypothesis that the recombination events in some cases might have occurred in the farm where the isolates of question were detected. However, in six cases, the putative vaccine origin parental strain was different from the vaccine strain used for immunization. The analysis on circulating and past European field strains resulted in similar findings, often resulting in a failure to firmly determine the true parental strains. The situation is further complicated by the lack of sequence information about the vaccine strains used in local flock immunization programs.

**Figure 4:**
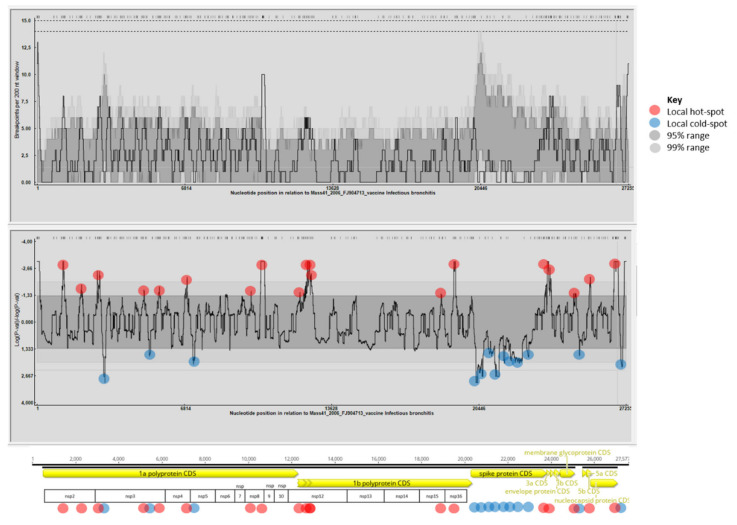
Recombination hot spots (red dots) were identified mostly in the regions coding for non-structural proteins (see multiple recombination hot spots in the nsp2, nsp3, nsp8, and nsp12 coding regions).

Recombination hot spots and cold spots were also determined for the data set (Figure 4). As reported by others in previous studies, recombination hot spots were identified along the entire genome [34,37], mostly in the regions coding for non-structural proteins. Interestingly, multiple recombination hot spots that affected individual protein coding regions were identified in the nsp2, nsp3, nsp8, and nsp12 coding regions. Such multiple hotspots were seen in genomic positions immediately upstream of the S gene, as well as in the nsp2, nsp3, and nsp16 genes, when analyzing several North American IBV strains [34]. In our study, regions with a limited number or no evidence of recombination were found intermittently near the 5′ and 3′ ends of the genome and in a large fragment of the S gene. This latter observation was particularly intriguing and deserves further explanation. The scarcity of recombination events within the S gene may somewhat contradict the data published in the literature, and it may be related to the data set we used in our analyses [38,39,40]. The random selection of study strains and the reference strains could introduce some unseen sampling bias; therefore, the interpretation of the obtained results needs caution. Nonetheless, we cannot exclude the possibility that biological characteristics (e.g., distinct selection pressure or distinct ability of strains to recombine in this region) could have played a role beyond this phenomenon.

It is tempting to speculate regarding the ecological and epidemiological conditions needed for recombination events to occur among various IBV strains. One scenario assigns a possible role to wild birds, which may harbor multiple, diverse Gammacoronaviruses [41,42]. However, only a few studies support that IBVs of chicken may infect a heterologous host [43,44,45]; thus, this hypothesis requires further evidence from genomic epidemiology studies conducted in wild birds. Furthermore, large-scale commercial chicken farming is conducted in closed facilities where direct contact between wild birds and chickens is unlikely. A more plausible scenario would link co-infections with genetically distinct IBV strains and subsequent recombination events among infecting viruses in the homologous host, chicken. Well-known horizontal and vertical transmission routes [9] are likely to aid the spread of recombinant strains in a flock, but human activity (including the movement of contaminated equipment and material) might also promote the dispersal of these IBVs over large distances. In this study, we found no evidence that any of the identified recombinant field-field or vaccine-field recombinant strains are able to spread over large distances, although the number of sequenced strains was limited. The extension of genomic epidemiology studies by collecting a large number of whole-genome sequence data would be needed to determine the long-term fitness of recombinant strains, as well as their transmissibility potential even among distant chicken flocks.

## 4. Conclusions

In this pilot genomic epidemiology study that aimed at investigating the genetic interaction(s) among European IBV strains, we sequenced 20 viral genomes. So far, due to budget constraints, the sample size was limited, and study strains represented only a portion of the whole IBV strain collection from the study period and area. Involving additional strains would have been useful to investigate the spatial and temporal patterns of recombination events in the field. Additionally, an increase in the number of identified recombination events could have been seen with lowering the strength of test parameters in the recombination analysis tools. A portion of vaccine strains with available GenBank records originated from historic IBV strains detected on other continents; therefore, their possible recombination history may have remained unseen, and their role in concurrent events involving field strains may be limited. Consistent with these limitations, the low number and the scattered geographical and temporal origin of IBV genome sequence data from Europe prevented us from depicting a more structured landscape of the genomic evolution of IBVs having circulated over the past decade on the continent.

Nonetheless, this study illustratively complements the growing body of evidence that IBVs are rapidly evolving coronaviruses, and genomic recombination is a major mechanism of genetic diversification of these members of the *Coronaviridae* family. We convincingly demonstrated that recombination occurs among different IBV genotypes and involves both field strains and field and vaccine IBV strains. Eighteen out of 20 study strains and all but two field strains with available GenBank records showed evidence of recombination. Furthermore, evidence showed that approximately 50% of vaccine strains were recombinant. Collectively, the present pilot study provides further information on the subject of the infection biology of IBV, an important coronavirus, with strong focus on viral genomic stability and recombination of the genomes of field and vaccine strains. This report provides useful support to the development of further improved diagnostic assays and of novel vaccine candidates, to combat IB in a more effective manner. 

## Figures and Tables

**Table 1:** Characteristics of infectious bronchitis virus (IBV) isolates whose genome sequence was determined in this study.

Isolate	Age (d)	Organ of Isolation	Country of Origin	Clinical Features/Pathology	Vaccination History	Genotype of Study Strain
D1656/2/11/BY/2011	31	CT	Belarus	Enteritis, hepatitis, cholecystitis, haemorrhages in intestinal track and caecal tonsils, atrophic bursa	ND	GI-19
D1478/2/2/10/GR/2010	40	CT	Greece	Respiratory disease (mortality 1% per day)	D0: H120; D15: H120	GI-13 (H120: GI-1)
D1719/1/11/GR/2011	26	CT	Greece	Mild respiratory disease, growth retardation	D1: Ark	GI-9 (Arkansas: GI-9)
D1760/2/2/11/GR/2011	29/40	T	Greece	Respiratory disease	D1: Ark; D18: 4/91	GI-19 (Ark: GI-9, 4/91: GI-13)
D2220/2/13/GR/2014	ND	CT	Greece	Respiratory, mortality	D1: H120; D18: 4/91	G1-19 (H120 and 4/91: GI-1)
D1561/8/11/HU/2011	7	CT	Hungary	ND	H120	GI-1 (H120: GI-1)
D1623/1/1/11/HU/2011	ND	T	Hungary	ND	ND	GI-19
D1871/1/1/12/HU/2012	ND	ND	Hungary	ND	ND	GI-1
11518-Kiskunmajsa/HU/2010	ND	ND	Hungary	Interstitial nephritis	ND	GI-19
211776-Imrehegy/HU/2011	28	K	Hungary	Interstitial nephritis (mortality 8%)	ND	GI-21
D2353/13/PL/2019	ND	ND	Poland	ND	ND	GI-13
D2002/2/12/PT/2012	35	T	Portugal	ND	D1: H120; D9: IB primer	GI-19 (H120: GI-1)
D1530/2/3/10/RO/2010	39	CT	Romania	Respiratory disease, swollen head, conjunctivitis (morbidity 50%, mortality 6-7%)	D1: Ma5; D9: 4/91	GI-13 (4/91: GI-13, Ma5: GI-1)
D1530/4/1/10/RO/2010	ND	K	Romania	ND	ND	GI-13
D1654/1/4/11/RO/2011	31	CT	Romania	Feed refusal syndrome, smaller body weight, nephritis, respiratory syndrome with tracheitis	D1: Ma5	GI-19 (Ma5: GI-1)
D1783/2/3/11/RO/2011	28	L, T, K	Romania	Dyspnoe, sneezing, tracheal congestion	D1: H120	GI-19 (H120: GI-1)
D1944/1/3/12/RO/2012	39	K	Romania	Respiratory, rales, sneezing, swollen head, white diarrhoea	D1: Ma5; D8: H120	GI-19 (Ma5 and H120: GI-1)
D2022/2/3/12/RO/2012	29	CT	Romania	Depression, ruffled feather, fever, withies diarrhoea	D6: IB 88	GI-19 (IB 88: GI-1)
D4000/3/17/RO/2017	39	CT	Romania	ND	ND	GI-21
D1617/3/11/UA/2011	28	T	Ukraine	Enteritis, tracheitis, nephritis	D0: IB primer; D10: H120	GI-19 (H120: GI-1)

ND, no data. D = day; age of birds at vaccination. K, kidney; CT, caecal tonsil; T, trachea; L, lung.

**Table 2:** IBV strains with an available genome sequence in GenBank.

Origin	Strain	Acc. No.	Origin	Strain	Acc. No.
Field isolate	CK/SWE/0658946/10	JQ088078	Vaccine strain	Massachusetts type	FJ904713, FJ904720-FJ904723, GQ504725, KY626045, MK728875
	gammaCoV/Ck/Poland/G225/2017	MK581208		Arkansas type	GQ504721 MH779856-MH779860
	gammaCoV/Ck/Poland/G103/2016	MK581207		Connecticut type	FJ904716-FJ904719 KF696629
	gammaCoV/Ck/Poland/G052/2016	KY047602		California type	FJ904714, FJ904715
	gammaCoV/Ck/Poland/548/2004	MK581205		Georgia type	GQ504723
	gammaCoV/Ck/Poland/255/1997	MK581204		L1148	KY933090
	gammaCoV/Ck/Poland/195/2012	MK581206		YX10	MF508703
	gammaCoV/Ck/Poland/162/1997	MK581203		VicS-v	KF460437
	gammaCoV/Ck/Poland/80/1989	MK581202		VicS-del	KF931628
	gammaCoV/Ck/Poland/79/1989	MK581201		BR-I	KY626044
	gammaCoV/Ck/Poland/78/1989	MK581200		SAIBK	DQ288927
	gammaCoV/Ck/Poland/74/2009	KT886454		1148-A	KY933089
	gammaCoV/Ck/Italy/I2022/13	KP780179		4/91	KF377577
	gammaCoV/AvCov/Ck/Italy/624I/96	MG021194		H52	EU817497
	B1648	KR231009		H120	FJ888351, GU393335
	IBVUkr27-11	KJ135013			

## Data Availability

Newly determined sequence data were deposited in GenBank (Accession Nos. MT984583-MT984602).

## Supplementary Materials

Supplementary materials can be found at https://www.mdpi.com/1999-4915/13/4/535/s1.
