# Supplementary material for: Recombination Events Shape the Genomic Evolution of Infectious Bronchitis Virus in Europe
Source: Viruses. 2021 Mar 24;13(4):535. doi: 10.3390/v13040535 (PMC8063831; doi:10.3390/v13040535)

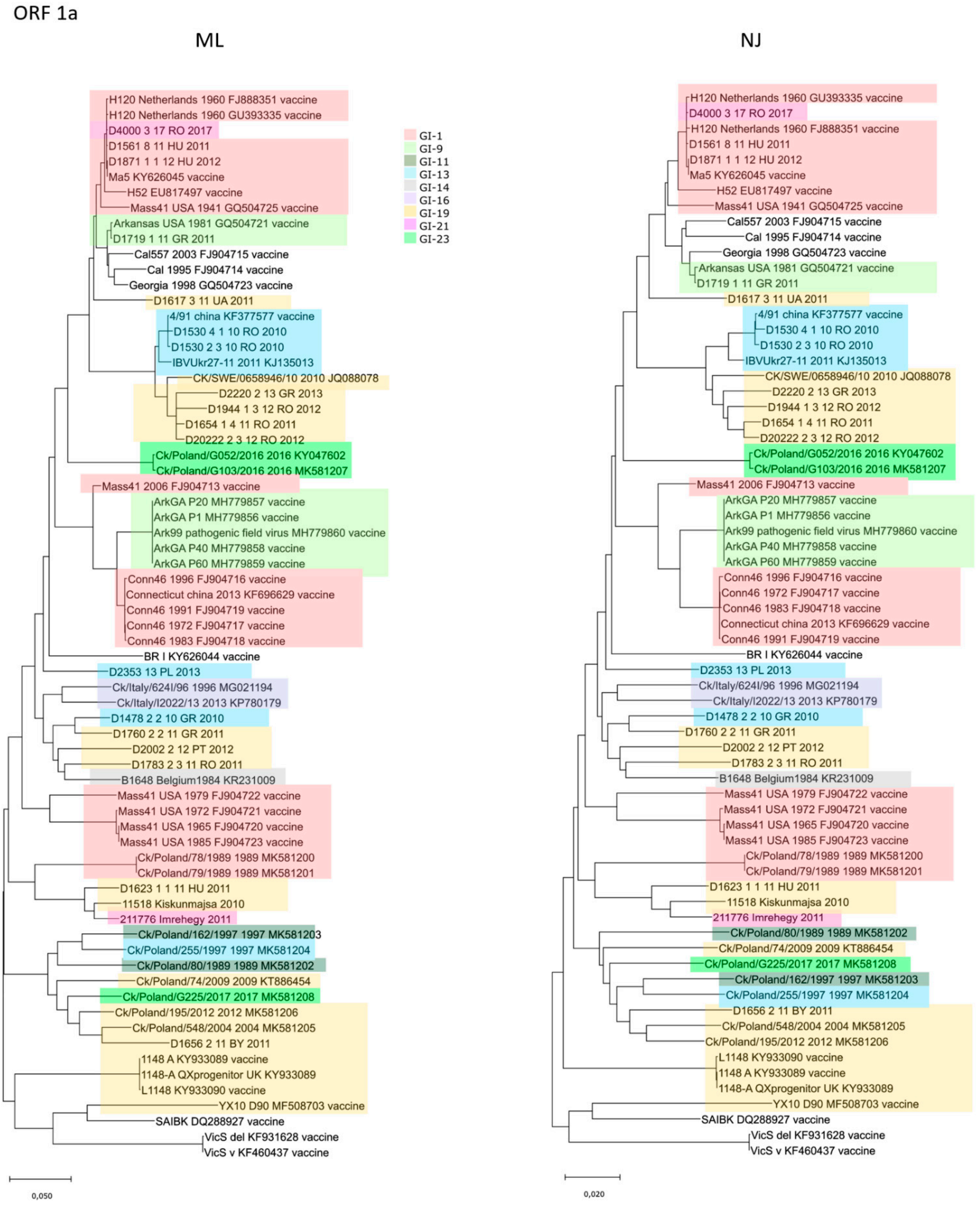
ORF 1a
ML
NJ
GI-1
GI-9
GI-11
GI-13
GI-14
GI-16
GI-19
GI-21
GI-23
H120 Netherlands 1960 FJ888351 vaccine
H120 Netherlands 1960 GU393335 vaccine
D4000 3 17 RO 2017
D1561 8 11 HU 2011
D1871 1 1 12 HU 2012
Ma5 KY626045 vaccine
H52 EU817497 vaccine
Mass41 USA 1941 GQ504725 vaccine
Arkansas USA 1981 GQ504721 vaccine
D1719 1 11 GR 2011
Cal557 2003 FJ904715 vaccine
Cal 1995 FJ904714 vaccine
Georgia 1998 GQ504723 vaccine
D1617 3 11 UA 2011
4/91 china KF377577 vaccine
D1530 4 1 10 RO 2010
D1530 2 3 10 RO 2010
IBVUkr27-11 2011 KJ135013
CK/SWE/0658946/10 2010 JQ088078
D2220 2 13 GR 2013
D1944 1 3 12 RO 2012
D1654 1 4 11 RO 2011
D20222 2 3 12 RO 2012
Ck/Poland/G052/2016 2016 KY047602
Ck/Poland/G103/2016 2016 MK581207
Mass41 2006 FJ904713 vaccine
ArkGA P20 MH779857 vaccine
ArkGA P1 MH779856 vaccine
Ark99 pathogenic field virus MH779860 vaccine
ArkGA P40 MH779858 vaccine
ArkGA P60 MH779859 vaccine
Conn46 1996 FJ904716 vaccine
Connecticut china 2013 KF696629 vaccine
Conn46 1991 FJ904719 vaccine
Conn46 1972 FJ904717 vaccine
Conn46 1983 FJ904718 vaccine
BR I KY626044 vaccine
D2353 13 PL 2013
Ck/Italy/624I/96 1996 MG021194
Ck/Italy/I2022/13 2013 KP780179
D1478 2 2 10 GR 2010
D1760 2 2 11 GR 2011
D2002 2 12 PT 2012
D1783 2 3 11 RO 2011
B1648 Belgium1984 KR231009
Mass41 USA 1979 FJ904722 vaccine
Mass41 USA 1972 FJ904721 vaccine
Mass41 USA 1965 FJ904720 vaccine
Mass41 USA 1985 FJ904723 vaccine
Ck/Poland/78/1989 1989 MK581200
Ck/Poland/79/1989 1989 MK581201
D1623 1 1 11 HU 2011
11518 Kiskunmajsa 2010
211776 Imrehegy 2011
Ck/Poland/162/1997 1997 MK581203
Ck/Poland/255/1997 1997 MK581204
Ck/Poland/80/1989 1989 MK581202
Ck/Poland/74/2009 2009 KT886454
Ck/Poland/G225/2017 2017 MK581208
Ck/Poland/195/2012 2012 MK581206
Ck/Poland/548/2004 2004 MK581205
D1656 2 11 BY 2011
1148 A KY933089 vaccine
1148-A QXprogenitor UK KY933089
L1148 KY933090 vaccine
YX10 D90 MF508703 vaccine
SAIBK DQ288927 vaccine
VicS del KF931628 vaccine
VicS v KF460437 vaccine
0,050
H120 Netherlands 1960 GU393335 vaccine
D4000 3 17 RO 2017
H120 Netherlands 1960 FJ888351 vaccine
D1561 8 11 HU 2011
D1871 1 1 12 HU 2012
Ma5 KY626045 vaccine
H52 EU817497 vaccine
Mass41 USA 1941 GQ504725 vaccine
Cal557 2003 FJ904715 vaccine
Cal 1995 FJ904714 vaccine
Georgia 1998 GQ504723 vaccine
Arkansas USA 1981 GQ504721 vaccine
D1719 1 11 GR 2011
D1617 3 11 UA 2011
4/91 china KF377577 vaccine
D1530 4 1 10 RO 2010
D1530 2 3 10 RO 2010
IBVUkr27-11 2011 KJ135013
CK/SWE/0658946/10 2010 JQ088078
D2220 2 13 GR 2013
D1944 1 3 12 RO 2012
D1654 1 4 11 RO 2011
D20222 2 3 12 RO 2012
Ck/Poland/G052/2016 2016 KY047602
Ck/Poland/G103/2016 2016 MK581207
Mass41 2006 FJ904713 vaccine
ArkGA P20 MH779857 vaccine
ArkGA P1 MH779856 vaccine
Ark99 pathogenic field virus MH779860 vaccine
ArkGA P40 MH779858 vaccine
ArkGA P60 MH779859 vaccine
Conn46 1996 FJ904716 vaccine
Conn46 1972 FJ904717 vaccine
Conn46 1983 FJ904718 vaccine
Connecticut china 2013 KF696629 vaccine
Conn46 1991 FJ904719 vaccine
BR I KY626044 vaccine
D2353 13 PL 2013
Ck/Italy/624I/96 1996 MG021194
Ck/Italy/I2022/13 2013 KP780179
D1478 2 2 10 GR 2010
D1760 2 2 11 GR 2011
D2002 2 12 PT 2012
D1783 2 3 11 RO 2011
B1648 Belgium1984 KR231009
Mass41 USA 1979 FJ904722 vaccine
Mass41 USA 1972 FJ904721 vaccine
Mass41 USA 1965 FJ904720 vaccine
Mass41 USA 1985 FJ904723 vaccine
Ck/Poland/78/1989 1989 MK581200
Ck/Poland/79/1989 1989 MK581201
D1623 1 1 11 HU 2011
11518 Kiskunmajsa 2010
211776 Imrehegy 2011
Ck/Poland/80/1989 1989 MK581202
Ck/Poland/74/2009 2009 KT886454
Ck/Poland/G225/2017 2017 MK581208
Ck/Poland/162/1997 1997 MK581203
Ck/Poland/255/1997 1997 MK581204
D1656 2 11 BY 2011
Ck/Poland/548/2004 2004 MK581205
Ck/Poland/195/2012 2012 MK581206
L1148 KY933090 vaccine
1148 A KY933089 vaccine
1148-A QXprogenitor UK KY933089
YX10 D90 MF508703 vaccine
SAIBK DQ288927 vaccine
VicS del KF931628 vaccine
VicS v KF460437 vaccine
0,020

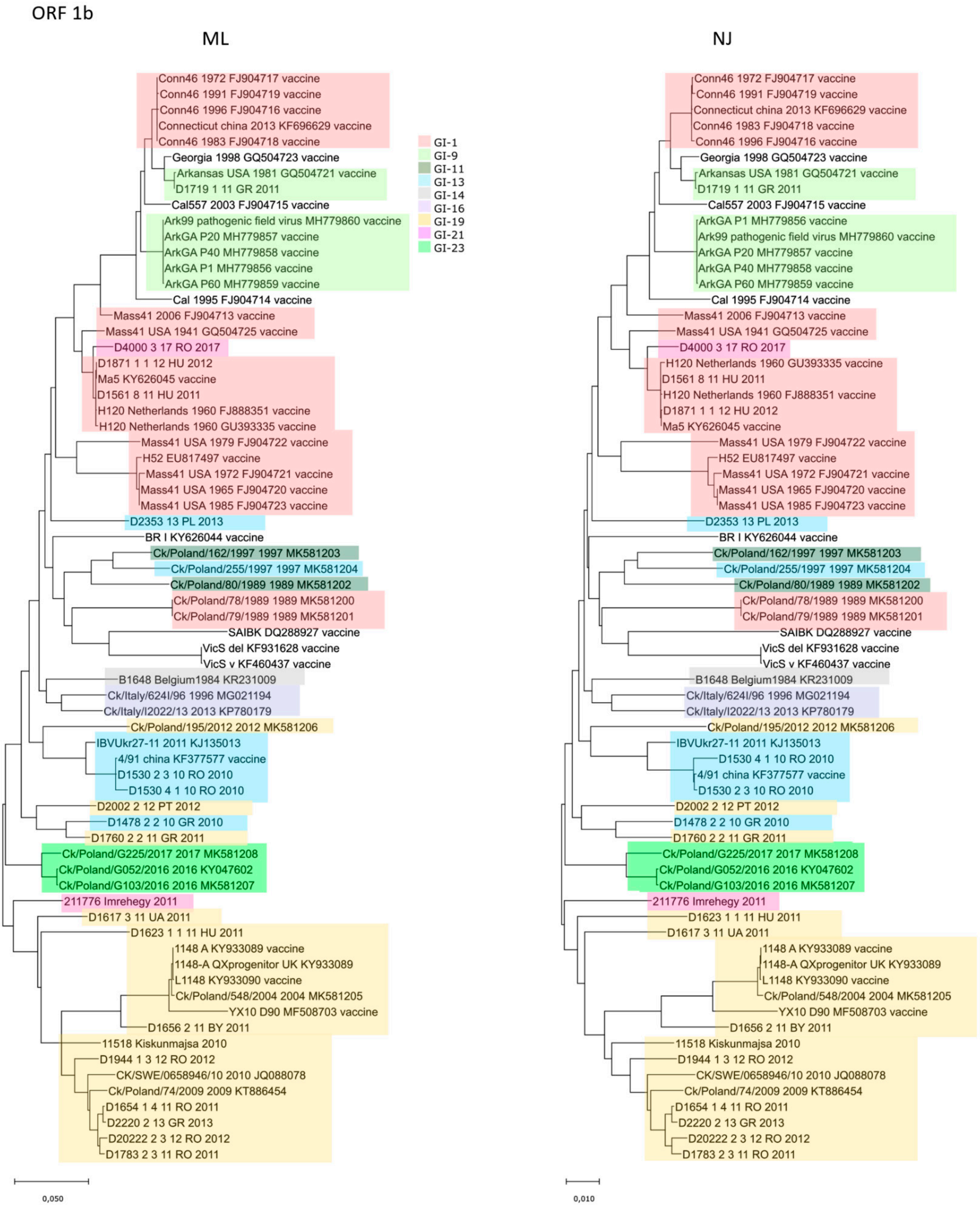
ORF 1b
ML
Conn46 1972 FJ904717 vaccine
Conn46 1991 FJ904719 vaccine
Conn46 1996 FJ904716 vaccine
Connecticut china 2013 KF696629 vaccine
Conn46 1983 FJ904718 vaccine
Georgia 1998 GQ504723 vaccine
Arkansas USA 1981 GQ504721 vaccine
D1719 1 11 GR 2011
Cal557 2003 FJ904715 vaccine
Ark99 pathogenic field virus MH779860 vaccine
ArkGA P20 MH779857 vaccine
ArkGA P40 MH779858 vaccine
ArkGA P1 MH779856 vaccine
ArkGA P60 MH779859 vaccine
Cal 1995 FJ904714 vaccine
Mass41 2006 FJ904713 vaccine
Mass41 USA 1941 GQ504725 vaccine
D4000 3 17 RO 2017
D1871 1 1 12 HU 2012
Ma5 KY626045 vaccine
D1561 8 11 HU 2011
H120 Netherlands 1960 FJ888351 vaccine
H120 Netherlands 1960 GU393335 vaccine
Mass41 USA 1979 FJ904722 vaccine
H52 EU817497 vaccine
Mass41 USA 1972 FJ904721 vaccine
Mass41 USA 1965 FJ904720 vaccine
Mass41 USA 1985 FJ904723 vaccine
D2353 13 PL 2013
BR I KY626044 vaccine
Ck/Poland/162/1997 1997 MK581203
Ck/Poland/255/1997 1997 MK581204
Ck/Poland/80/1989 1989 MK581202
Ck/Poland/78/1989 1989 MK581200
Ck/Poland/79/1989 1989 MK581201
SAIBK DQ288927 vaccine
VicS del KF931628 vaccine
VicS v KF460437 vaccine
B1648 Belgium1984 KR231009
Ck/Italy/624I/96 1996 MG021194
Ck/Italy/I2022/13 2013 KP780179
Ck/Poland/195/2012 2012 MK581206
IBVUkr27-11 2011 KJ135013
4/91 china KF377577 vaccine
D1530 2 3 10 RO 2010
D1530 4 1 10 RO 2010
D2002 2 12 PT 2012
D1478 2 2 10 GR 2010
D1760 2 2 11 GR 2011
Ck/Poland/G225/2017 2017 MK581208
Ck/Poland/G052/2016 2016 KY047602
Ck/Poland/G103/2016 2016 MK581207
211776 Imrehegy 2011
D1617 3 11 UA 2011
D1623 1 1 11 HU 2011
1148 A KY933089 vaccine
1148-A QXprogenitor UK KY933089
L1148 KY933090 vaccine
Ck/Poland/548/2004 2004 MK581205
YX10 D90 MF508703 vaccine
D1656 2 11 BY 2011
11518 Kiskunmajsa 2010
D1944 1 3 12 RO 2012
CK/SWE/0658946/10 2010 JQ088078
Ck/Poland/74/2009 2009 KT886454
D1654 1 4 11 RO 2011
D2220 2 13 GR 2013
D20222 2 3 12 RO 2012
D1783 2 3 11 RO 2011
0,050
GI-1
GI-9
GI-11
GI-13
GI-14
GI-16
GI-19
GI-21
GI-23
NJ
Conn46 1972 FJ904717 vaccine
Conn46 1991 FJ904719 vaccine
Connecticut china 2013 KF696629 vaccine
Conn46 1983 FJ904718 vaccine
Conn46 1996 FJ904716 vaccine
Georgia 1998 GQ504723 vaccine
Arkansas USA 1981 GQ504721 vaccine
D1719 1 11 GR 2011
Cal557 2003 FJ904715 vaccine
ArkGA P1 MH779856 vaccine
Ark99 pathogenic field virus MH779860 vaccine
ArkGA P20 MH779857 vaccine
ArkGA P40 MH779858 vaccine
ArkGA P60 MH779859 vaccine
Cal 1995 FJ904714 vaccine
Mass41 2006 FJ904713 vaccine
Mass41 USA 1941 GQ504725 vaccine
D4000 3 17 RO 2017
H120 Netherlands 1960 GU393335 vaccine
D1561 8 11 HU 2011
H120 Netherlands 1960 FJ888351 vaccine
D1871 1 1 12 HU 2012
Ma5 KY626045 vaccine
Mass41 USA 1979 FJ904722 vaccine
H52 EU817497 vaccine
Mass41 USA 1972 FJ904721 vaccine
Mass41 USA 1965 FJ904720 vaccine
Mass41 USA 1985 FJ904723 vaccine
D2353 13 PL 2013
BR I KY626044 vaccine
Ck/Poland/162/1997 1997 MK581203
Ck/Poland/255/1997 1997 MK581204
Ck/Poland/80/1989 1989 MK581202
Ck/Poland/78/1989 1989 MK581200
Ck/Poland/79/1989 1989 MK581201
SAIBK DQ288927 vaccine
VicS del KF931628 vaccine
VicS v KF460437 vaccine
B1648 Belgium1984 KR231009
Ck/Italy/624I/96 1996 MG021194
Ck/Italy/I2022/13 2013 KP780179
Ck/Poland/195/2012 2012 MK581206
IBVUkr27-11 2011 KJ135013
D1530 4 1 10 RO 2010
4/91 china KF377577 vaccine
D1530 2 3 10 RO 2010
D2002 2 12 PT 2012
D1478 2 2 10 GR 2010
D1760 2 2 11 GR 2011
Ck/Poland/G225/2017 2017 MK581208
Ck/Poland/G052/2016 2016 KY047602
Ck/Poland/G103/2016 2016 MK581207
211776 Imrehegy 2011
D1623 1 1 11 HU 2011
D1617 3 11 UA 2011
1148 A KY933089 vaccine
1148-A QXprogenitor UK KY933089
L1148 KY933090 vaccine
Ck/Poland/548/2004 2004 MK581205
YX10 D90 MF508703 vaccine
D1656 2 11 BY 2011
11518 Kiskunmajsa 2010
D1944 1 3 12 RO 2012
CK/SWE/0658946/10 2010 JQ088078
Ck/Poland/74/2009 2009 KT886454
D1654 1 4 11 RO 2011
D2220 2 13 GR 2013
D20222 2 3 12 RO 2012
D1783 2 3 11 RO 2011
0,010

ORF 3a

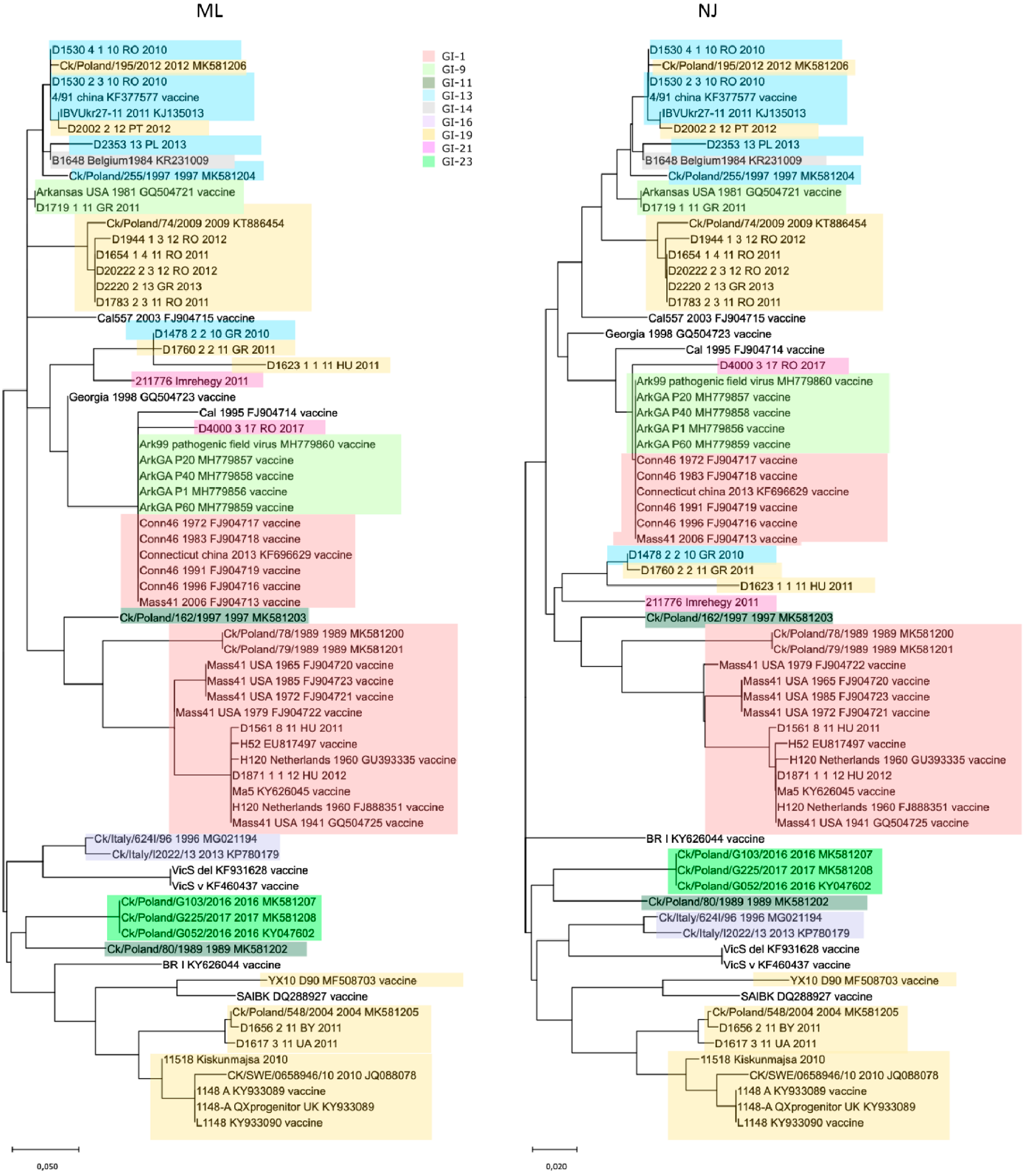
ML
NJ
GI-1
GI-9
GI-11
GI-13
GI-14
GI-16
GI-19
GI-21
GI-23
D1530 4 1 10 RO 2010
Ck/Poland/195/2012 2012 MK581206
D1530 2 3 10 RO 2010
4/91 china KF377577 vaccine
IBVUkr27-11 2011 KJ135013
D2002 2 12 PT 2012
D2353 13 PL 2013
B1648 Belgium1984 KR231009
Ck/Poland/255/1997 1997 MK581204
Arkansas USA 1981 GQ504721 vaccine
D1719 1 11 GR 2011
Ck/Poland/74/2009 2009 KT886454
D1944 1 3 12 RO 2012
D1654 1 4 11 RO 2011
D20222 2 3 12 RO 2012
D2220 2 13 GR 2013
D1783 2 3 11 RO 2011
Cal557 2003 FJ904715 vaccine
D1478 2 2 10 GR 2010
D1760 2 2 11 GR 2011
D1623 1 1 11 HU 2011
211776 Imrehegy 2011
Georgia 1998 GQ504723 vaccine
Cal 1995 FJ904714 vaccine
D4000 3 17 RO 2017
Ark99 pathogenic field virus MH779860 vaccine
ArkGA P20 MH779857 vaccine
ArkGA P40 MH779858 vaccine
ArkGA P1 MH779856 vaccine
ArkGA P60 MH779859 vaccine
Conn46 1972 FJ904717 vaccine
Conn46 1983 FJ904718 vaccine
Connecticut china 2013 KF696629 vaccine
Conn46 1991 FJ904719 vaccine
Conn46 1996 FJ904716 vaccine
Mass41 2006 FJ904713 vaccine
Ck/Poland/162/1997 1997 MK581203
Ck/Poland/78/1989 1989 MK581200
Ck/Poland/79/1989 1989 MK581201
Mass41 USA 1965 FJ904720 vaccine
Mass41 USA 1985 FJ904723 vaccine
Mass41 USA 1972 FJ904721 vaccine
Mass41 USA 1979 FJ904722 vaccine
D1561 8 11 HU 2011
H52 EU817497 vaccine
H120 Netherlands 1960 GU393335 vaccine
D1871 1 1 12 HU 2012
Ma5 KY626045 vaccine
H120 Netherlands 1960 FJ888351 vaccine
Mass41 USA 1941 GQ504725 vaccine
Ck/Italy/624I/96 1996 MG021194
Ck/Italy/I2022/13 2013 KP780179
VicS del KF931628 vaccine
VicS v KF460437 vaccine
Ck/Poland/G103/2016 2016 MK581207
Ck/Poland/G225/2017 2017 MK581208
Ck/Poland/G052/2016 2016 KY047602
Ck/Poland/80/1989 1989 MK581202
BR I KY626044 vaccine
YX10 D90 MF508703 vaccine
SAIBK DQ288927 vaccine
Ck/Poland/548/2004 2004 MK581205
D1656 2 11 BY 2011
D1617 3 11 UA 2011
11518 Kiskunmajsa 2010
CK/SWE/0658946/10 2010 JQ088078
1148 A KY933089 vaccine
1148-A QXprogenitor UK KY933089
L1148 KY933090 vaccine
0,050
D1530 4 1 10 RO 2010
Ck/Poland/195/2012 2012 MK581206
D1530 2 3 10 RO 2010
4/91 china KF377577 vaccine
IBVUkr27-11 2011 KJ135013
D2002 2 12 PT 2012
D2353 13 PL 2013
B1648 Belgium1984 KR231009
Ck/Poland/255/1997 1997 MK581204
Arkansas USA 1981 GQ504721 vaccine
D1719 1 11 GR 2011
Ck/Poland/74/2009 2009 KT886454
D1944 1 3 12 RO 2012
D1654 1 4 11 RO 2011
D20222 2 3 12 RO 2012
D2220 2 13 GR 2013
D1783 2 3 11 RO 2011
Cal557 2003 FJ904715 vaccine
Georgia 1998 GQ504723 vaccine
Cal 1995 FJ904714 vaccine
D4000 3 17 RO 2017
Ark99 pathogenic field virus MH779860 vaccine
ArkGA P20 MH779857 vaccine
ArkGA P40 MH779858 vaccine
ArkGA P1 MH779856 vaccine
ArkGA P60 MH779859 vaccine
Conn46 1972 FJ904717 vaccine
Conn46 1983 FJ904718 vaccine
Connecticut china 2013 KF696629 vaccine
Conn46 1991 FJ904719 vaccine
Conn46 1996 FJ904716 vaccine
Mass41 2006 FJ904713 vaccine
D1478 2 2 10 GR 2010
D1760 2 2 11 GR 2011
D1623 1 1 11 HU 2011
211776 Imrehegy 2011
Ck/Poland/162/1997 1997 MK581203
Ck/Poland/78/1989 1989 MK581200
Ck/Poland/79/1989 1989 MK581201
Mass41 USA 1979 FJ904722 vaccine
Mass41 USA 1965 FJ904720 vaccine
Mass41 USA 1985 FJ904723 vaccine
Mass41 USA 1972 FJ904721 vaccine
D1561 8 11 HU 2011
H52 EU817497 vaccine
H120 Netherlands 1960 GU393335 vaccine
D1871 1 1 12 HU 2012
Ma5 KY626045 vaccine
H120 Netherlands 1960 FJ888351 vaccine
Mass41 USA 1941 GQ504725 vaccine
BR I KY626044 vaccine
Ck/Poland/G103/2016 2016 MK581207
Ck/Poland/G225/2017 2017 MK581208
Ck/Poland/G052/2016 2016 KY047602
Ck/Poland/80/1989 1989 MK581202
Ck/Italy/624I/96 1996 MG021194
Ck/Italy/I2022/13 2013 KP780179
VicS del KF931628 vaccine
VicS v KF460437 vaccine
YX10 D90 MF508703 vaccine
SAIBK DQ288927 vaccine
Ck/Poland/548/2004 2004 MK581205
D1656 2 11 BY 2011
D1617 3 11 UA 2011
11518 Kiskunmajsa 2010
CK/SWE/0658946/10 2010 JQ088078
1148 A KY933089 vaccine
1148-A QXprogenitor UK KY933089
L1148 KY933090 vaccine
0,020

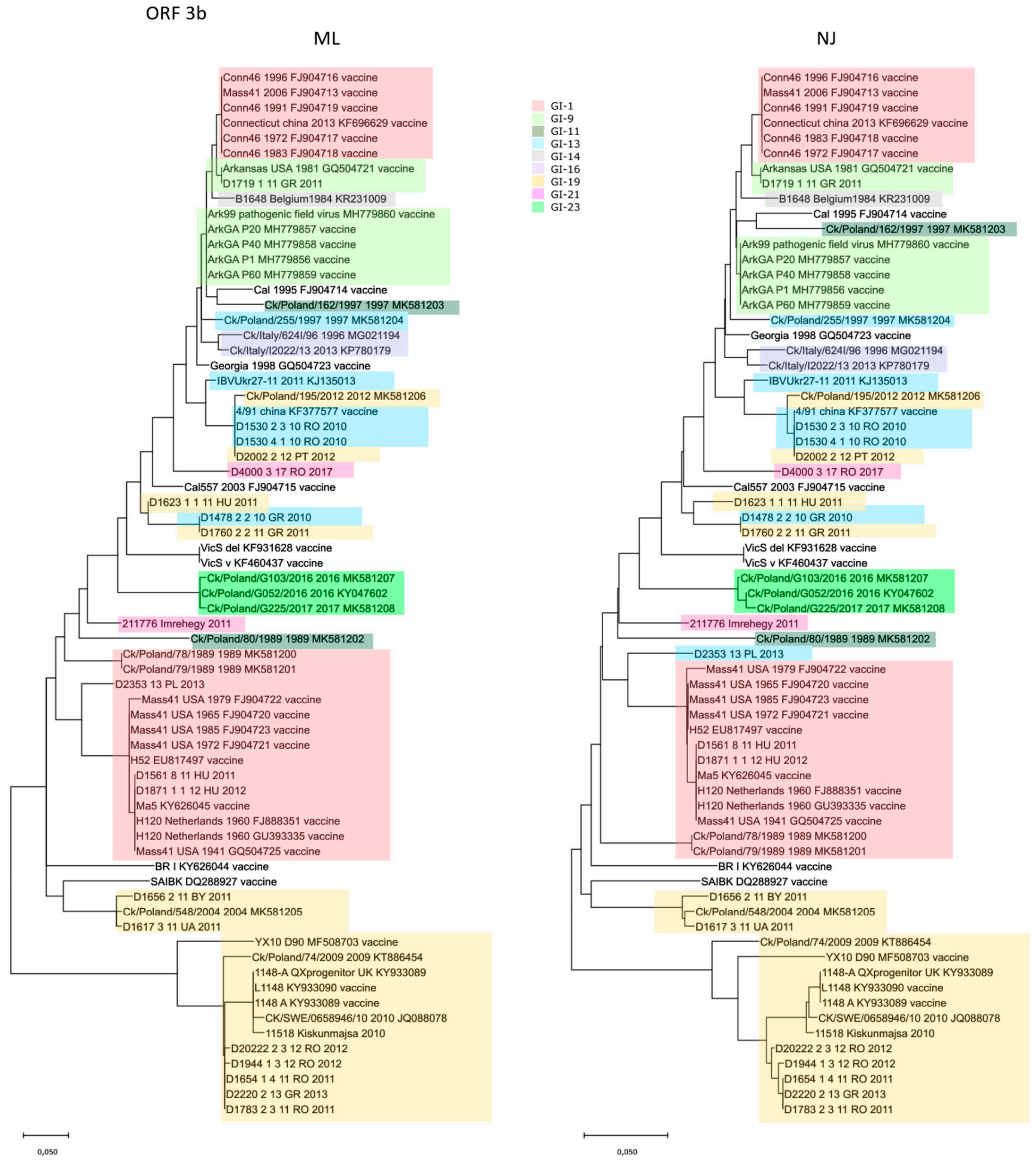
ORF 3b
ML
NJ
GI-1
GI-9
GI-11
GI-13
GI-14
GI-16
GI-19
GI-21
GI-23
Conn46 1996 FJ904716 vaccine
Mass41 2006 FJ904713 vaccine
Conn46 1991 FJ904719 vaccine
Connecticut china 2013 KF696629 vaccine
Conn46 1972 FJ904717 vaccine
Conn46 1983 FJ904718 vaccine
Arkansas USA 1981 GQ504721 vaccine
D1719 1 11 GR 2011
B1648 Belgium1984 KR231009
Ark99 pathogenic field virus MH779860 vaccine
ArkGA P20 MH779857 vaccine
ArkGA P40 MH779858 vaccine
ArkGA P1 MH779856 vaccine
ArkGA P60 MH779859 vaccine
Cal 1995 FJ904714 vaccine
Ck/Poland/162/1997 1997 MK581203
Ck/Poland/255/1997 1997 MK581204
Ck/Italy/624I/96 1996 MG021194
Ck/Italy/I2022/13 2013 KP780179
Georgia 1998 GQ504723 vaccine
IBVUkr27-11 2011 KJ135013
Ck/Poland/195/2012 2012 MK581206
4/91 china KF377577 vaccine
D1530 2 3 10 RO 2010
D1530 4 1 10 RO 2010
D2002 2 12 PT 2012
D4000 3 17 RO 2017
Cal557 2003 FJ904715 vaccine
D1623 1 1 11 HU 2011
D1478 2 2 10 GR 2010
D1760 2 2 11 GR 2011
VicS del KF931628 vaccine
VicS v KF460437 vaccine
Ck/Poland/G103/2016 2016 MK581207
Ck/Poland/G052/2016 2016 KY047602
Ck/Poland/G225/2017 2017 MK581208
211776 Imrehegy 2011
Ck/Poland/80/1989 1989 MK581202
Ck/Poland/78/1989 1989 MK581200
Ck/Poland/79/1989 1989 MK581201
D2353 13 PL 2013
Mass41 USA 1979 FJ904722 vaccine
Mass41 USA 1965 FJ904720 vaccine
Mass41 USA 1985 FJ904723 vaccine
Mass41 USA 1972 FJ904721 vaccine
H52 EU817497 vaccine
D1561 8 11 HU 2011
D1871 1 1 12 HU 2012
Ma5 KY626045 vaccine
H120 Netherlands 1960 FJ888351 vaccine
H120 Netherlands 1960 GU393335 vaccine
Mass41 USA 1941 GQ504725 vaccine
BR I KY626044 vaccine
SAIBK DQ288927 vaccine
D1656 2 11 BY 2011
Ck/Poland/548/2004 2004 MK581205
D1617 3 11 UA 2011
YX10 D90 MF508703 vaccine
Ck/Poland/74/2009 2009 KT886454
1148-A QXprogenitor UK KY933089
L1148 KY933090 vaccine
1148 A KY933089 vaccine
CK/SWE/0658946/10 2010 JQ088078
11518 Kiskunmajsa 2010
D20222 2 3 12 RO 2012
D1944 1 3 12 RO 2012
D1654 1 4 11 RO 2011
D2220 2 13 GR 2013
D1783 2 3 11 RO 2011
0,050
Conn46 1996 FJ904716 vaccine
Mass41 2006 FJ904713 vaccine
Conn46 1991 FJ904719 vaccine
Connecticut china 2013 KF696629 vaccine
Conn46 1983 FJ904718 vaccine
Conn46 1972 FJ904717 vaccine
Arkansas USA 1981 GQ504721 vaccine
D1719 1 11 GR 2011
B1648 Belgium1984 KR231009
Cal 1995 FJ904714 vaccine
Ck/Poland/162/1997 1997 MK581203
Ark99 pathogenic field virus MH779860 vaccine
ArkGA P20 MH779857 vaccine
ArkGA P40 MH779858 vaccine
ArkGA P1 MH779856 vaccine
ArkGA P60 MH779859 vaccine
Ck/Poland/255/1997 1997 MK581204
Georgia 1998 GQ504723 vaccine
Ck/Italy/624I/96 1996 MG021194
Ck/Italy/I2022/13 2013 KP780179
IBVUkr27-11 2011 KJ135013
Ck/Poland/195/2012 2012 MK581206
4/91 china KF377577 vaccine
D1530 2 3 10 RO 2010
D1530 4 1 10 RO 2010
D2002 2 12 PT 2012
D4000 3 17 RO 2017
Cal557 2003 FJ904715 vaccine
D1623 1 1 11 HU 2011
D1478 2 2 10 GR 2010
D1760 2 2 11 GR 2011
VicS del KF931628 vaccine
VicS v KF460437 vaccine
Ck/Poland/G103/2016 2016 MK581207
Ck/Poland/G052/2016 2016 KY047602
Ck/Poland/G225/2017 2017 MK581208
211776 Imrehegy 2011
Ck/Poland/80/1989 1989 MK581202
D2353 13 PL 2013
Mass41 USA 1979 FJ904722 vaccine
Mass41 USA 1965 FJ904720 vaccine
Mass41 USA 1985 FJ904723 vaccine
Mass41 USA 1972 FJ904721 vaccine
H52 EU817497 vaccine
D1561 8 11 HU 2011
D1871 1 1 12 HU 2012
Ma5 KY626045 vaccine
H120 Netherlands 1960 FJ888351 vaccine
H120 Netherlands 1960 GU393335 vaccine
Mass41 USA 1941 GQ504725 vaccine
Ck/Poland/78/1989 1989 MK581200
Ck/Poland/79/1989 1989 MK581201
BR I KY626044 vaccine
SAIBK DQ288927 vaccine
D1656 2 11 BY 2011
Ck/Poland/548/2004 2004 MK581205
D1617 3 11 UA 2011
Ck/Poland/74/2009 2009 KT886454
YX10 D90 MF508703 vaccine
1148-A QXprogenitor UK KY933089
L1148 KY933090 vaccine
1148 A KY933089 vaccine
CK/SWE/0658946/10 2010 JQ088078
11518 Kiskunmajsa 2010
D20222 2 3 12 RO 2012
D1944 1 3 12 RO 2012
D1654 1 4 11 RO 2011
D2220 2 13 GR 2013
D1783 2 3 11 RO 2011
0,050

ORF 3c (envelope protein coding gene)

ML NJ

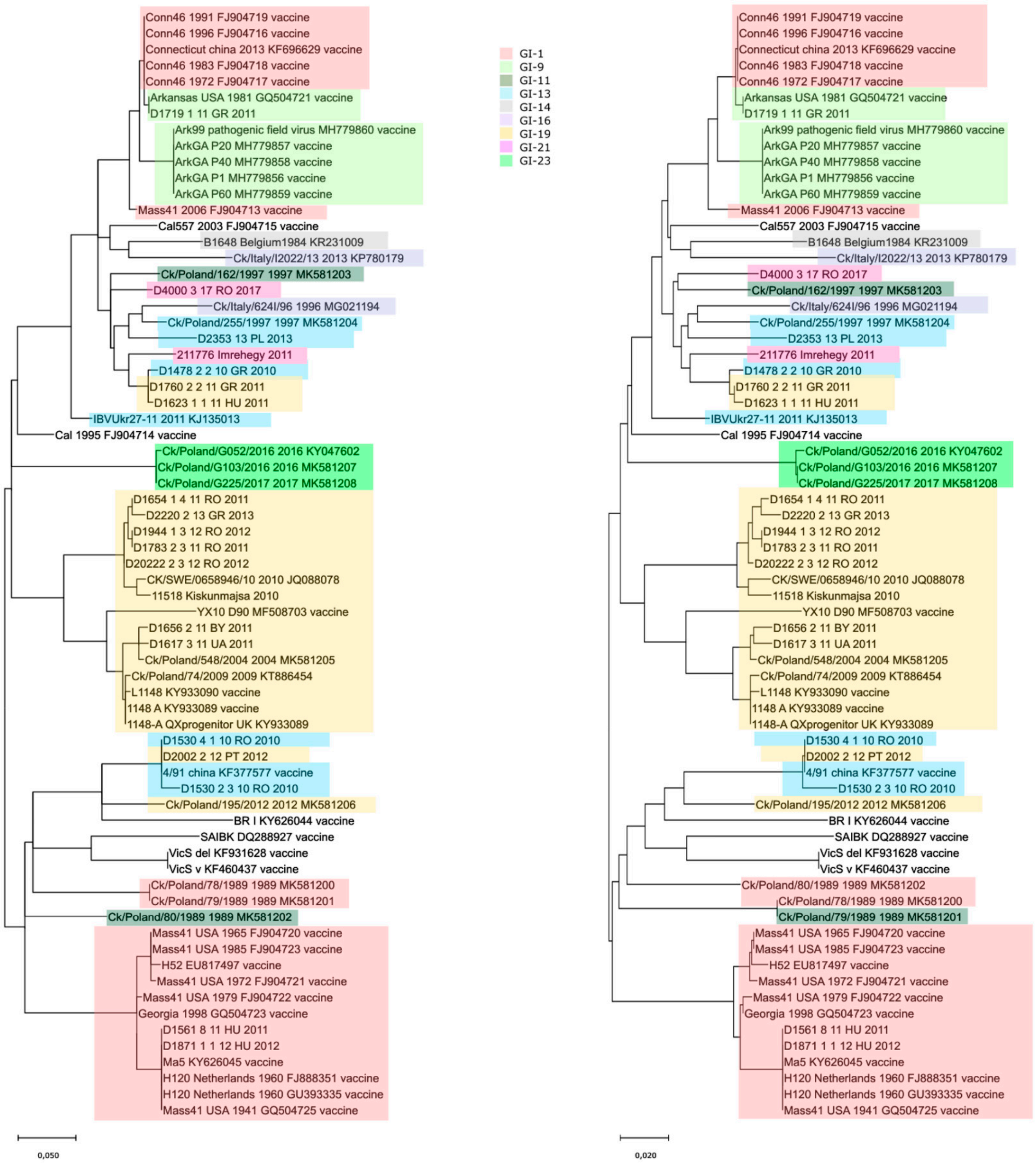

ORF 4a (membrane protein coding gene)

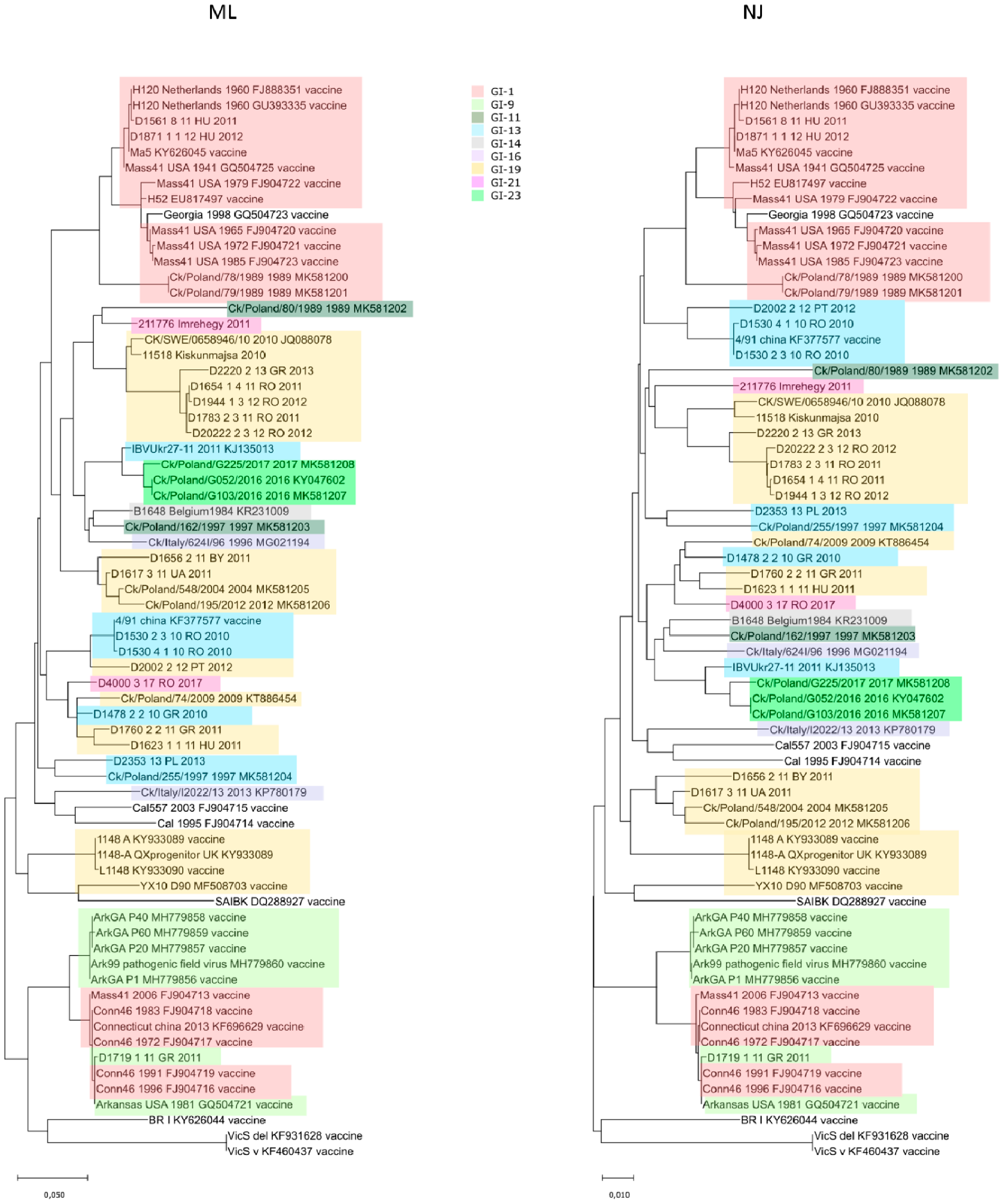

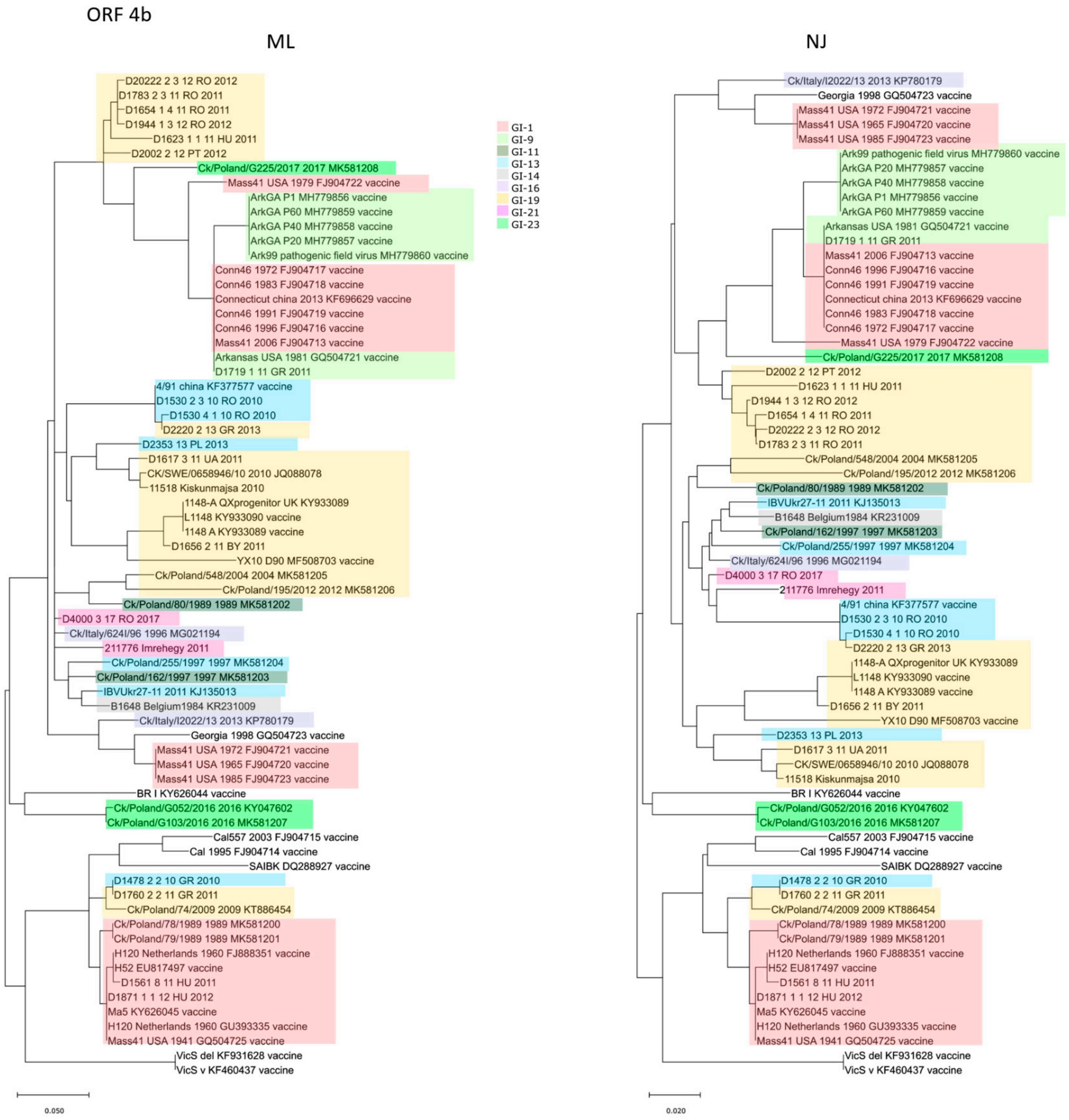
ORF 4b
ML
D20222 2 3 12 RO 2012
D1783 2 3 11 RO 2011
D1654 1 4 11 RO 2011
D1944 1 3 12 RO 2012
D1623 1 1 11 HU 2011
D2002 2 12 PT 2012
Ck/Poland/G225/2017 2017 MK581208
Mass41 USA 1979 FJ904722 vaccine
ArkGA P1 MH779856 vaccine
ArkGA P60 MH779859 vaccine
ArkGA P40 MH779858 vaccine
ArkGA P20 MH779857 vaccine
Ark99 pathogenic field virus MH779860 vaccine
Conn46 1972 FJ904717 vaccine
Conn46 1983 FJ904718 vaccine
Connecticut china 2013 KF696629 vaccine
Conn46 1991 FJ904719 vaccine
Conn46 1996 FJ904716 vaccine
Mass41 2006 FJ904713 vaccine
Arkansas USA 1981 GQ504721 vaccine
D1719 1 11 GR 2011
4/91 china KF377577 vaccine
D1530 2 3 10 RO 2010
D1530 4 1 10 RO 2010
D2220 2 13 GR 2013
D2353 13 PL 2013
D1617 3 11 UA 2011
CK/SWE/0658946/10 2010 JQ088078
11518 Kiskunmajsa 2010
1148-A QXprogenitor UK KY933089
L1148 KY933090 vaccine
1148 A KY933089 vaccine
D1656 2 11 BY 2011
YX10 D90 MF508703 vaccine
Ck/Poland/548/2004 2004 MK581205
Ck/Poland/195/2012 2012 MK581206
Ck/Poland/80/1989 1989 MK581202
D4000 3 17 RO 2017
Ck/Italy/624I/96 1996 MG021194
211776 Imrehegy 2011
Ck/Poland/255/1997 1997 MK581204
Ck/Poland/162/1997 1997 MK581203
IBVUkr27-11 2011 KJ135013
B1648 Belgium1984 KR231009
Ck/Italy/I2022/13 2013 KP780179
Georgia 1998 GQ504723 vaccine
Mass41 USA 1972 FJ904721 vaccine
Mass41 USA 1965 FJ904720 vaccine
Mass41 USA 1985 FJ904723 vaccine
BR I KY626044 vaccine
Ck/Poland/G052/2016 2016 KY047602
Ck/Poland/G103/2016 2016 MK581207
Cal557 2003 FJ904715 vaccine
Cal 1995 FJ904714 vaccine
SAIBK DQ288927 vaccine
D1478 2 2 10 GR 2010
D1760 2 2 11 GR 2011
Ck/Poland/74/2009 2009 KT886454
Ck/Poland/78/1989 1989 MK581200
Ck/Poland/79/1989 1989 MK581201
H120 Netherlands 1960 FJ888351 vaccine
H52 EU817497 vaccine
D1561 8 11 HU 2011
D1871 1 1 12 HU 2012
Ma5 KY626045 vaccine
H120 Netherlands 1960 GU393335 vaccine
Mass41 USA 1941 GQ504725 vaccine
VicS del KF931628 vaccine
VicS v KF460437 vaccine
0.050
GI-1
GI-9
GI-11
GI-13
GI-14
GI-16
GI-19
GI-21
GI-23
NJ
Ck/Italy/I2022/13 2013 KP780179
Georgia 1998 GQ504723 vaccine
Mass41 USA 1972 FJ904721 vaccine
Mass41 USA 1965 FJ904720 vaccine
Mass41 USA 1985 FJ904723 vaccine
Ark99 pathogenic field virus MH779860 vaccine
ArkGA P20 MH779857 vaccine
ArkGA P40 MH779858 vaccine
ArkGA P1 MH779856 vaccine
ArkGA P60 MH779859 vaccine
Arkansas USA 1981 GQ504721 vaccine
D1719 1 11 GR 2011
Mass41 2006 FJ904713 vaccine
Conn46 1996 FJ904716 vaccine
Conn46 1991 FJ904719 vaccine
Connecticut china 2013 KF696629 vaccine
Conn46 1983 FJ904718 vaccine
Conn46 1972 FJ904717 vaccine
Mass41 USA 1979 FJ904722 vaccine
Ck/Poland/G225/2017 2017 MK581208
D2002 2 12 PT 2012
D1623 1 1 11 HU 2011
D1944 1 3 12 RO 2012
D1654 1 4 11 RO 2011
D20222 2 3 12 RO 2012
D1783 2 3 11 RO 2011
Ck/Poland/548/2004 2004 MK581205
Ck/Poland/195/2012 2012 MK581206
Ck/Poland/80/1989 1989 MK581202
IBVUkr27-11 2011 KJ135013
B1648 Belgium1984 KR231009
Ck/Poland/162/1997 1997 MK581203
Ck/Poland/255/1997 1997 MK581204
Ck/Italy/624I/96 1996 MG021194
D4000 3 17 RO 2017
211776 Imrehegy 2011
4/91 china KF377577 vaccine
D1530 2 3 10 RO 2010
D1530 4 1 10 RO 2010
D2220 2 13 GR 2013
1148-A QXprogenitor UK KY933089
L1148 KY933090 vaccine
1148 A KY933089 vaccine
D1656 2 11 BY 2011
YX10 D90 MF508703 vaccine
D2353 13 PL 2013
D1617 3 11 UA 2011
CK/SWE/0658946/10 2010 JQ088078
11518 Kiskunmajsa 2010
BR I KY626044 vaccine
Ck/Poland/G052/2016 2016 KY047602
Ck/Poland/G103/2016 2016 MK581207
Cal557 2003 FJ904715 vaccine
Cal 1995 FJ904714 vaccine
SAIBK DQ288927 vaccine
D1478 2 2 10 GR 2010
D1760 2 2 11 GR 2011
Ck/Poland/74/2009 2009 KT886454
Ck/Poland/78/1989 1989 MK581200
Ck/Poland/79/1989 1989 MK581201
H120 Netherlands 1960 FJ888351 vaccine
H52 EU817497 vaccine
D1561 8 11 HU 2011
D1871 1 1 12 HU 2012
Ma5 KY626045 vaccine
H120 Netherlands 1960 GU393335 vaccine
Mass41 USA 1941 GQ504725 vaccine
VicS del KF931628 vaccine
VicS v KF460437 vaccine
0.020

ORF 4c

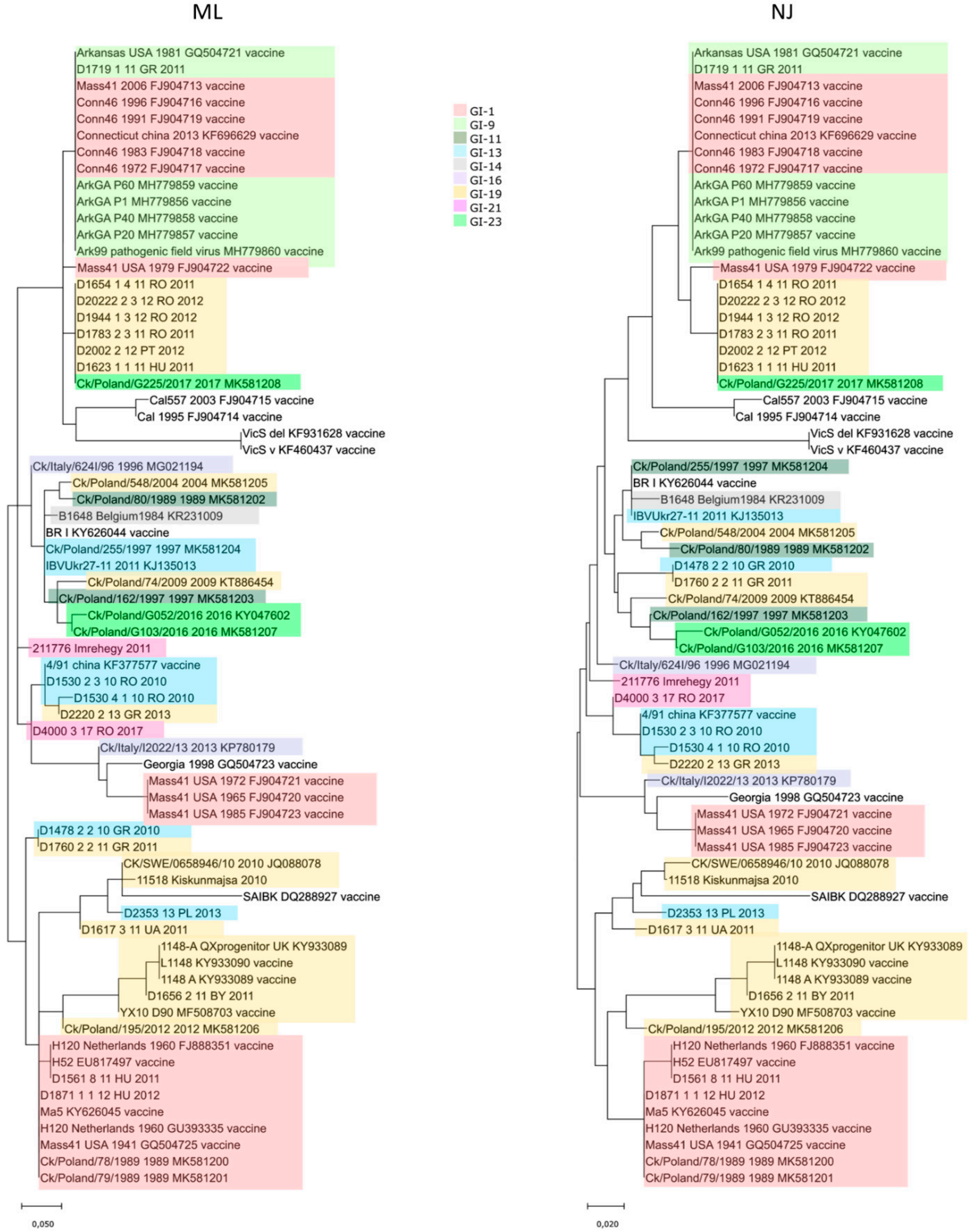
ML
NJ
GI-1
GI-9
GI-11
GI-13
GI-14
GI-16
GI-19
GI-21
GI-23
Arkansas USA 1981 GQ504721 vaccine
D1719 1 11 GR 2011
Mass41 2006 FJ904713 vaccine
Conn46 1996 FJ904716 vaccine
Conn46 1991 FJ904719 vaccine
Connecticut china 2013 KF696629 vaccine
Conn46 1983 FJ904718 vaccine
Conn46 1972 FJ904717 vaccine
ArkGA P60 MH779859 vaccine
ArkGA P1 MH779856 vaccine
ArkGA P40 MH779858 vaccine
ArkGA P20 MH779857 vaccine
Ark99 pathogenic field virus MH779860 vaccine
Mass41 USA 1979 FJ904722 vaccine
D1654 1 4 11 RO 2011
D20222 2 3 12 RO 2012
D1944 1 3 12 RO 2012
D1783 2 3 11 RO 2011
D2002 2 12 PT 2012
D1623 1 1 11 HU 2011
Ck/Poland/G225/2017 2017 MK581208
Cal557 2003 FJ904715 vaccine
Cal 1995 FJ904714 vaccine
VicS del KF931628 vaccine
VicS v KF460437 vaccine
Ck/Italy/624I/96 1996 MG021194
Ck/Poland/548/2004 2004 MK581205
Ck/Poland/80/1989 1989 MK581202
B1648 Belgium1984 KR231009
BR I KY626044 vaccine
Ck/Poland/255/1997 1997 MK581204
IBVUkr27-11 2011 KJ135013
Ck/Poland/74/2009 2009 KT886454
Ck/Poland/162/1997 1997 MK581203
Ck/Poland/G052/2016 2016 KY047602
Ck/Poland/G103/2016 2016 MK581207
211776 Imrehegy 2011
4/91 china KF377577 vaccine
D1530 2 3 10 RO 2010
D1530 4 1 10 RO 2010
D2220 2 13 GR 2013
D4000 3 17 RO 2017
Ck/Italy/I2022/13 2013 KP780179
Georgia 1998 GQ504723 vaccine
Mass41 USA 1972 FJ904721 vaccine
Mass41 USA 1965 FJ904720 vaccine
Mass41 USA 1985 FJ904723 vaccine
D1478 2 2 10 GR 2010
D1760 2 2 11 GR 2011
CK/SWE/0658946/10 2010 JQ088078
11518 Kiskunmajsa 2010
SAIBK DQ288927 vaccine
D2353 13 PL 2013
D1617 3 11 UA 2011
1148-A QXprogenitor UK KY933089
L1148 KY933090 vaccine
1148 A KY933089 vaccine
D1656 2 11 BY 2011
YX10 D90 MF508703 vaccine
Ck/Poland/195/2012 2012 MK581206
H120 Netherlands 1960 FJ888351 vaccine
H52 EU817497 vaccine
D1561 8 11 HU 2011
D1871 1 1 12 HU 2012
Ma5 KY626045 vaccine
H120 Netherlands 1960 GU393335 vaccine
Mass41 USA 1941 GQ504725 vaccine
Ck/Poland/78/1989 1989 MK581200
Ck/Poland/79/1989 1989 MK581201
0,050
Arkansas USA 1981 GQ504721 vaccine
D1719 1 11 GR 2011
Mass41 2006 FJ904713 vaccine
Conn46 1996 FJ904716 vaccine
Conn46 1991 FJ904719 vaccine
Connecticut china 2013 KF696629 vaccine
Conn46 1983 FJ904718 vaccine
Conn46 1972 FJ904717 vaccine
ArkGA P60 MH779859 vaccine
ArkGA P1 MH779856 vaccine
ArkGA P40 MH779858 vaccine
ArkGA P20 MH779857 vaccine
Ark99 pathogenic field virus MH779860 vaccine
Mass41 USA 1979 FJ904722 vaccine
D1654 1 4 11 RO 2011
D20222 2 3 12 RO 2012
D1944 1 3 12 RO 2012
D1783 2 3 11 RO 2011
D2002 2 12 PT 2012
D1623 1 1 11 HU 2011
Ck/Poland/G225/2017 2017 MK581208
Cal557 2003 FJ904715 vaccine
Cal 1995 FJ904714 vaccine
VicS del KF931628 vaccine
VicS v KF460437 vaccine
Ck/Poland/255/1997 1997 MK581204
BR I KY626044 vaccine
B1648 Belgium1984 KR231009
IBVUkr27-11 2011 KJ135013
Ck/Poland/548/2004 2004 MK581205
Ck/Poland/80/1989 1989 MK581202
D1478 2 2 10 GR 2010
D1760 2 2 11 GR 2011
Ck/Poland/74/2009 2009 KT886454
Ck/Poland/162/1997 1997 MK581203
Ck/Poland/G052/2016 2016 KY047602
Ck/Poland/G103/2016 2016 MK581207
Ck/Italy/624I/96 1996 MG021194
211776 Imrehegy 2011
D4000 3 17 RO 2017
4/91 china KF377577 vaccine
D1530 2 3 10 RO 2010
D1530 4 1 10 RO 2010
D2220 2 13 GR 2013
Ck/Italy/I2022/13 2013 KP780179
Georgia 1998 GQ504723 vaccine
Mass41 USA 1972 FJ904721 vaccine
Mass41 USA 1965 FJ904720 vaccine
Mass41 USA 1985 FJ904723 vaccine
CK/SWE/0658946/10 2010 JQ088078
11518 Kiskunmajsa 2010
SAIBK DQ288927 vaccine
D2353 13 PL 2013
D1617 3 11 UA 2011
1148-A QXprogenitor UK KY933089
L1148 KY933090 vaccine
1148 A KY933089 vaccine
D1656 2 11 BY 2011
YX10 D90 MF508703 vaccine
Ck/Poland/195/2012 2012 MK581206
H120 Netherlands 1960 FJ888351 vaccine
H52 EU817497 vaccine
D1561 8 11 HU 2011
D1871 1 1 12 HU 2012
Ma5 KY626045 vaccine
H120 Netherlands 1960 GU393335 vaccine
Mass41 USA 1941 GQ504725 vaccine
Ck/Poland/78/1989 1989 MK581200
Ck/Poland/79/1989 1989 MK581201
0,020

ORF 5a

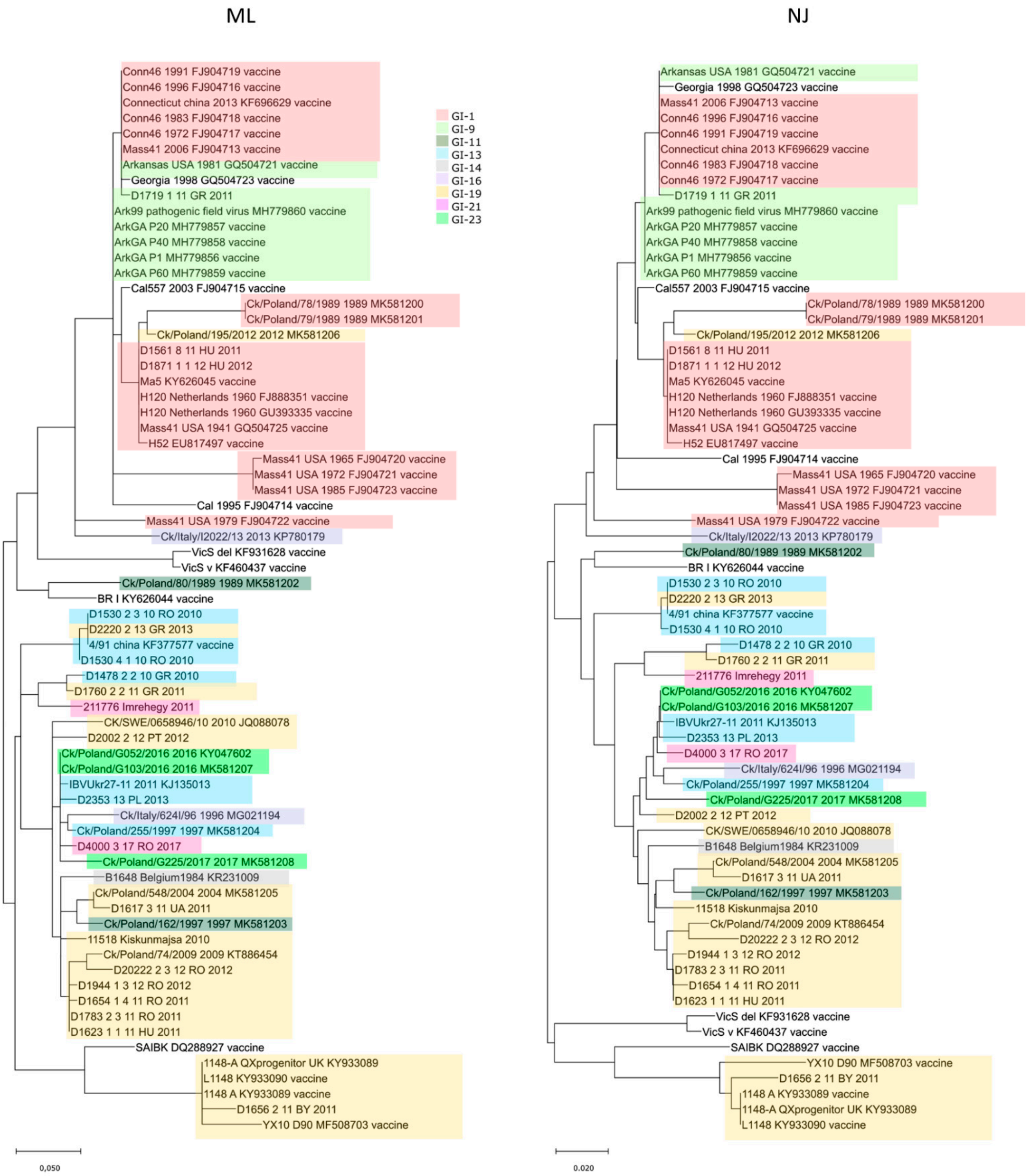
ML
NJ
GI-1
GI-9
GI-11
GI-13
GI-14
GI-16
GI-19
GI-21
GI-23
Conn46 1991 FJ904719 vaccine
Conn46 1996 FJ904716 vaccine
Connecticut china 2013 KF696629 vaccine
Conn46 1983 FJ904718 vaccine
Conn46 1972 FJ904717 vaccine
Mass41 2006 FJ904713 vaccine
Arkansas USA 1981 GQ504721 vaccine
Georgia 1998 GQ504723 vaccine
D1719 1 11 GR 2011
Ark99 pathogenic field virus MH779860 vaccine
ArkGA P20 MH779857 vaccine
ArkGA P40 MH779858 vaccine
ArkGA P1 MH779856 vaccine
ArkGA P60 MH779859 vaccine
Cal557 2003 FJ904715 vaccine
Ck/Poland/78/1989 1989 MK581200
Ck/Poland/79/1989 1989 MK581201
Ck/Poland/195/2012 2012 MK581206
D1561 8 11 HU 2011
D1871 1 1 12 HU 2012
Ma5 KY626045 vaccine
H120 Netherlands 1960 FJ888351 vaccine
H120 Netherlands 1960 GU393335 vaccine
Mass41 USA 1941 GQ504725 vaccine
H52 EU817497 vaccine
Mass41 USA 1965 FJ904720 vaccine
Mass41 USA 1972 FJ904721 vaccine
Mass41 USA 1985 FJ904723 vaccine
Cal 1995 FJ904714 vaccine
Mass41 USA 1979 FJ904722 vaccine
Ck/Italy/I2022/13 2013 KP780179
VicS del KF931628 vaccine
VicS v KF460437 vaccine
Ck/Poland/80/1989 1989 MK581202
BR I KY626044 vaccine
D1530 2 3 10 RO 2010
D2220 2 13 GR 2013
4/91 china KF377577 vaccine
D1530 4 1 10 RO 2010
D1478 2 2 10 GR 2010
D1760 2 2 11 GR 2011
211776 Imrehegy 2011
CK/SWE/0658946/10 2010 JQ088078
D2002 2 12 PT 2012
Ck/Poland/G052/2016 2016 KY047602
Ck/Poland/G103/2016 2016 MK581207
IBVUkr27-11 2011 KJ135013
D2353 13 PL 2013
Ck/Italy/624I/96 1996 MG021194
Ck/Poland/255/1997 1997 MK581204
D4000 3 17 RO 2017
Ck/Poland/G225/2017 2017 MK581208
B1648 Belgium1984 KR231009
Ck/Poland/548/2004 2004 MK581205
D1617 3 11 UA 2011
Ck/Poland/162/1997 1997 MK581203
11518 Kiskunmajsa 2010
Ck/Poland/74/2009 2009 KT886454
D20222 2 3 12 RO 2012
D1944 1 3 12 RO 2012
D1654 1 4 11 RO 2011
D1783 2 3 11 RO 2011
D1623 1 1 11 HU 2011
SAIBK DQ288927 vaccine
1148-A QXprogenitor UK KY933089
L1148 KY933090 vaccine
1148 A KY933089 vaccine
D1656 2 11 BY 2011
YX10 D90 MF508703 vaccine
0,050
Arkansas USA 1981 GQ504721 vaccine
Georgia 1998 GQ504723 vaccine
Mass41 2006 FJ904713 vaccine
Conn46 1996 FJ904716 vaccine
Conn46 1991 FJ904719 vaccine
Connecticut china 2013 KF696629 vaccine
Conn46 1983 FJ904718 vaccine
Conn46 1972 FJ904717 vaccine
D1719 1 11 GR 2011
Ark99 pathogenic field virus MH779860 vaccine
ArkGA P20 MH779857 vaccine
ArkGA P40 MH779858 vaccine
ArkGA P1 MH779856 vaccine
ArkGA P60 MH779859 vaccine
Cal557 2003 FJ904715 vaccine
Ck/Poland/78/1989 1989 MK581200
Ck/Poland/79/1989 1989 MK581201
Ck/Poland/195/2012 2012 MK581206
D1561 8 11 HU 2011
D1871 1 1 12 HU 2012
Ma5 KY626045 vaccine
H120 Netherlands 1960 FJ888351 vaccine
H120 Netherlands 1960 GU393335 vaccine
Mass41 USA 1941 GQ504725 vaccine
H52 EU817497 vaccine
Cal 1995 FJ904714 vaccine
Mass41 USA 1965 FJ904720 vaccine
Mass41 USA 1972 FJ904721 vaccine
Mass41 USA 1985 FJ904723 vaccine
Mass41 USA 1979 FJ904722 vaccine
Ck/Italy/I2022/13 2013 KP780179
Ck/Poland/80/1989 1989 MK581202
BR I KY626044 vaccine
D1530 2 3 10 RO 2010
D2220 2 13 GR 2013
4/91 china KF377577 vaccine
D1530 4 1 10 RO 2010
D1478 2 2 10 GR 2010
D1760 2 2 11 GR 2011
211776 Imrehegy 2011
Ck/Poland/G052/2016 2016 KY047602
Ck/Poland/G103/2016 2016 MK581207
IBVUkr27-11 2011 KJ135013
D2353 13 PL 2013
D4000 3 17 RO 2017
Ck/Italy/624I/96 1996 MG021194
Ck/Poland/255/1997 1997 MK581204
Ck/Poland/G225/2017 2017 MK581208
D2002 2 12 PT 2012
CK/SWE/0658946/10 2010 JQ088078
B1648 Belgium1984 KR231009
Ck/Poland/548/2004 2004 MK581205
D1617 3 11 UA 2011
Ck/Poland/162/1997 1997 MK581203
11518 Kiskunmajsa 2010
Ck/Poland/74/2009 2009 KT886454
D20222 2 3 12 RO 2012
D1944 1 3 12 RO 2012
D1783 2 3 11 RO 2011
D1654 1 4 11 RO 2011
D1623 1 1 11 HU 2011
VicS del KF931628 vaccine
VicS v KF460437 vaccine
SAIBK DQ288927 vaccine
YX10 D90 MF508703 vaccine
D1656 2 11 BY 2011
1148 A KY933089 vaccine
1148-A QXprogenitor UK KY933089
L1148 KY933090 vaccine
0.020

ORF 5b

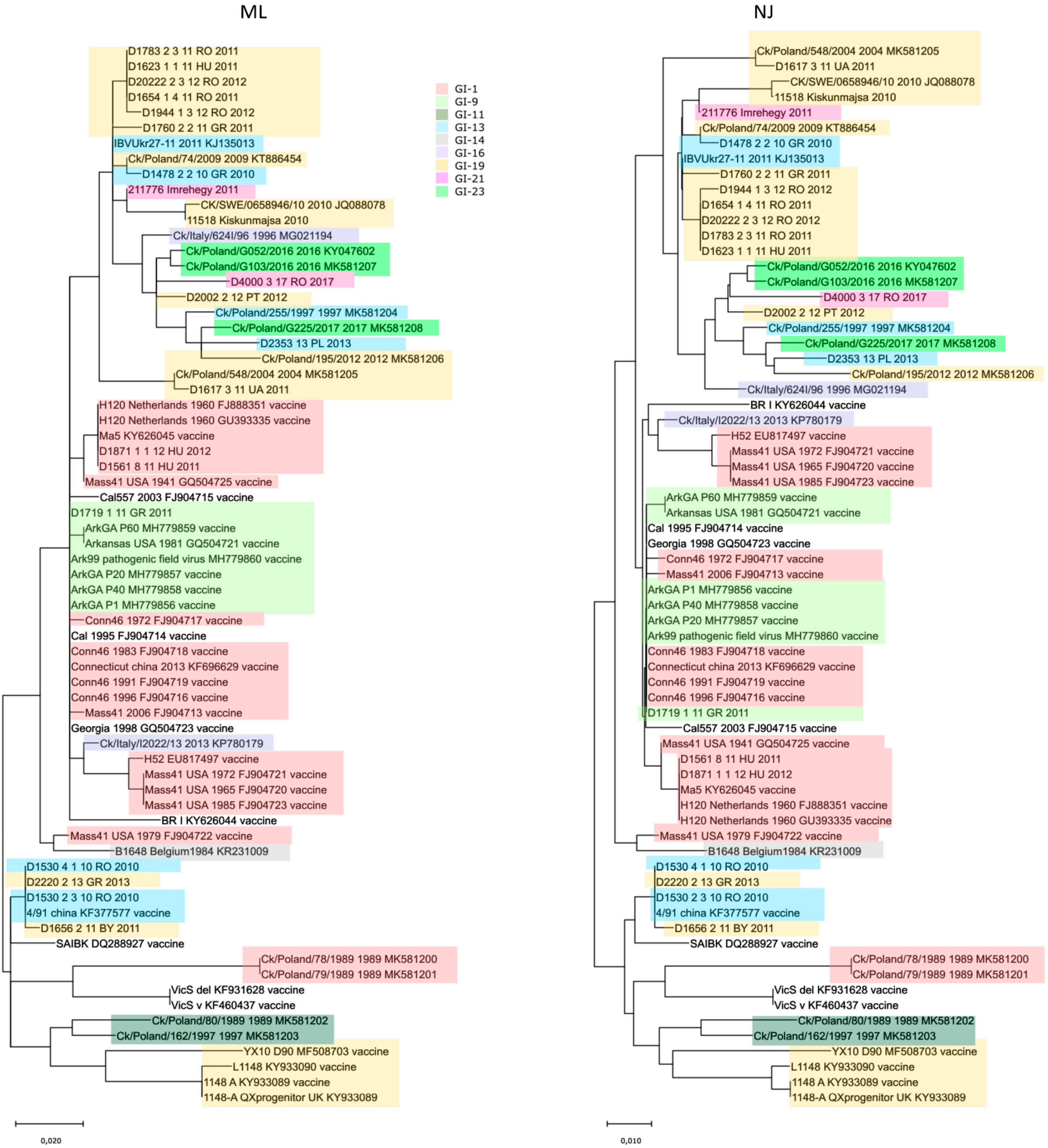
ML
NJ
GI-1
GI-9
GI-11
GI-13
GI-14
GI-16
GI-19
GI-21
GI-23
D1783 2 3 11 RO 2011
D1623 1 1 11 HU 2011
D20222 2 3 12 RO 2012
D1654 1 4 11 RO 2011
D1944 1 3 12 RO 2012
D1760 2 2 11 GR 2011
IBVUkr27-11 2011 KJ135013
Ck/Poland/74/2009 2009 KT886454
D1478 2 2 10 GR 2010
211776 Imrehegy 2011
CK/SWE/0658946/10 2010 JQ088078
11518 Kiskunmajsa 2010
Ck/Italy/6241/96 1996 MG021194
Ck/Poland/G052/2016 2016 KY047602
Ck/Poland/G103/2016 2016 MK581207
D4000 3 17 RO 2017
D2002 2 12 PT 2012
Ck/Poland/255/1997 1997 MK581204
Ck/Poland/G225/2017 2017 MK581208
D2353 13 PL 2013
Ck/Poland/195/2012 2012 MK581206
Ck/Poland/548/2004 2004 MK581205
D1617 3 11 UA 2011
H120 Netherlands 1960 FJ888351 vaccine
H120 Netherlands 1960 GU393335 vaccine
Ma5 KY626045 vaccine
D1871 1 1 12 HU 2012
D1561 8 11 HU 2011
Mass41 USA 1941 GQ504725 vaccine
Cal557 2003 FJ904715 vaccine
D1719 1 11 GR 2011
ArkGA P60 MH779859 vaccine
Arkansas USA 1981 GQ504721 vaccine
Ark99 pathogenic field virus MH779860 vaccine
ArkGA P20 MH779857 vaccine
ArkGA P40 MH779858 vaccine
ArkGA P1 MH779856 vaccine
Conn46 1972 FJ904717 vaccine
Cal 1995 FJ904714 vaccine
Conn46 1983 FJ904718 vaccine
Connecticut china 2013 KF696629 vaccine
Conn46 1991 FJ904719 vaccine
Conn46 1996 FJ904716 vaccine
Mass41 2006 FJ904713 vaccine
Georgia 1998 GQ504723 vaccine
Ck/Italy/I2022/13 2013 KP780179
H52 EU817497 vaccine
Mass41 USA 1972 FJ904721 vaccine
Mass41 USA 1965 FJ904720 vaccine
Mass41 USA 1985 FJ904723 vaccine
BR I KY626044 vaccine
Mass41 USA 1979 FJ904722 vaccine
B1648 Belgium1984 KR231009
D1530 4 1 10 RO 2010
D2220 2 13 GR 2013
D1530 2 3 10 RO 2010
4/91 china KF377577 vaccine
D1656 2 11 BY 2011
SAIBK DQ288927 vaccine
Ck/Poland/78/1989 1989 MK581200
Ck/Poland/79/1989 1989 MK581201
VicS del KF931628 vaccine
VicS v KF460437 vaccine
Ck/Poland/80/1989 1989 MK581202
Ck/Poland/162/1997 1997 MK581203
YX10 D90 MF508703 vaccine
L1148 KY933090 vaccine
1148 A KY933089 vaccine
1148-A QXprogenitor UK KY933089
0,020
0,010

Nucleocapsid protein coding gene

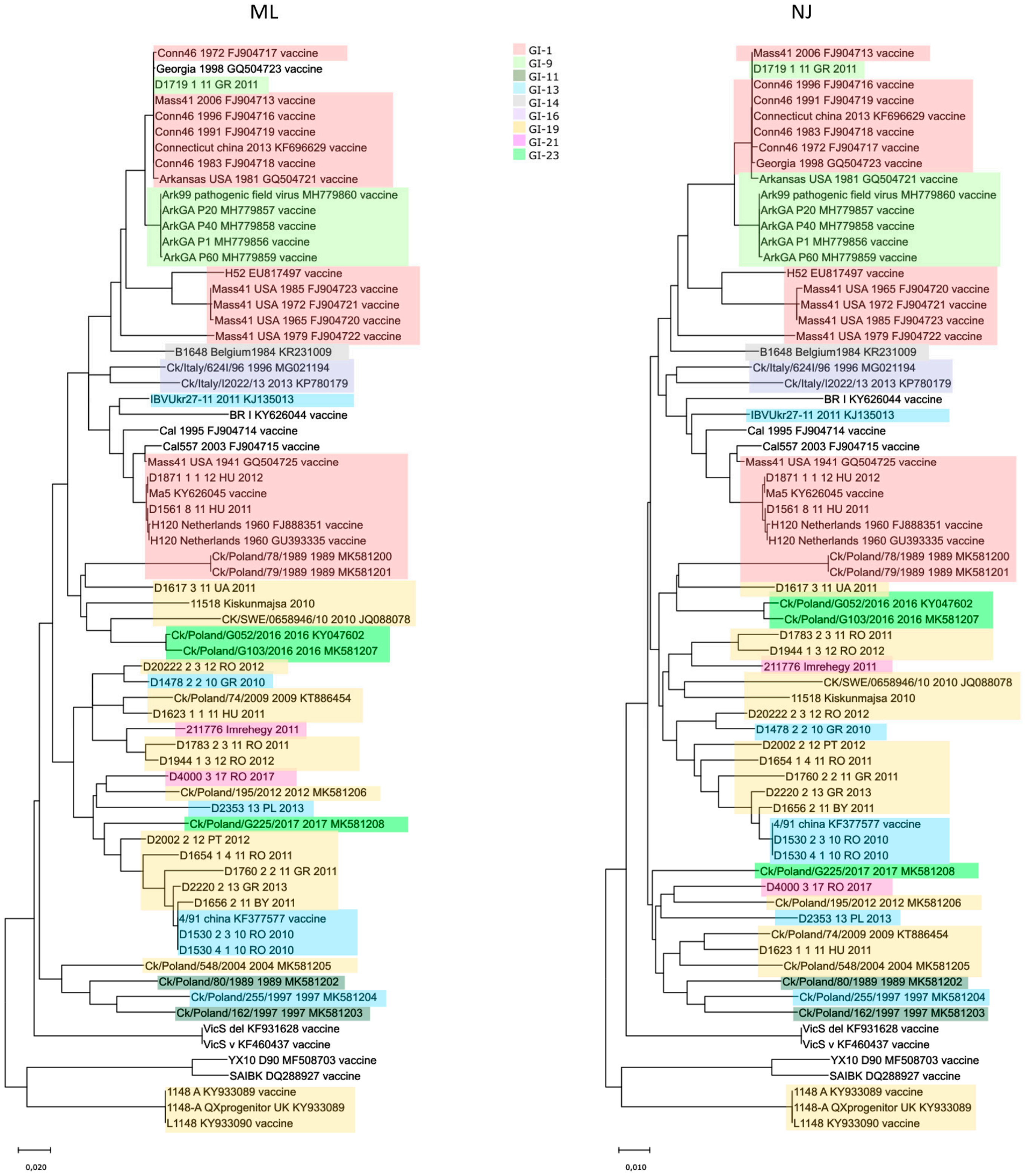

S1 gene

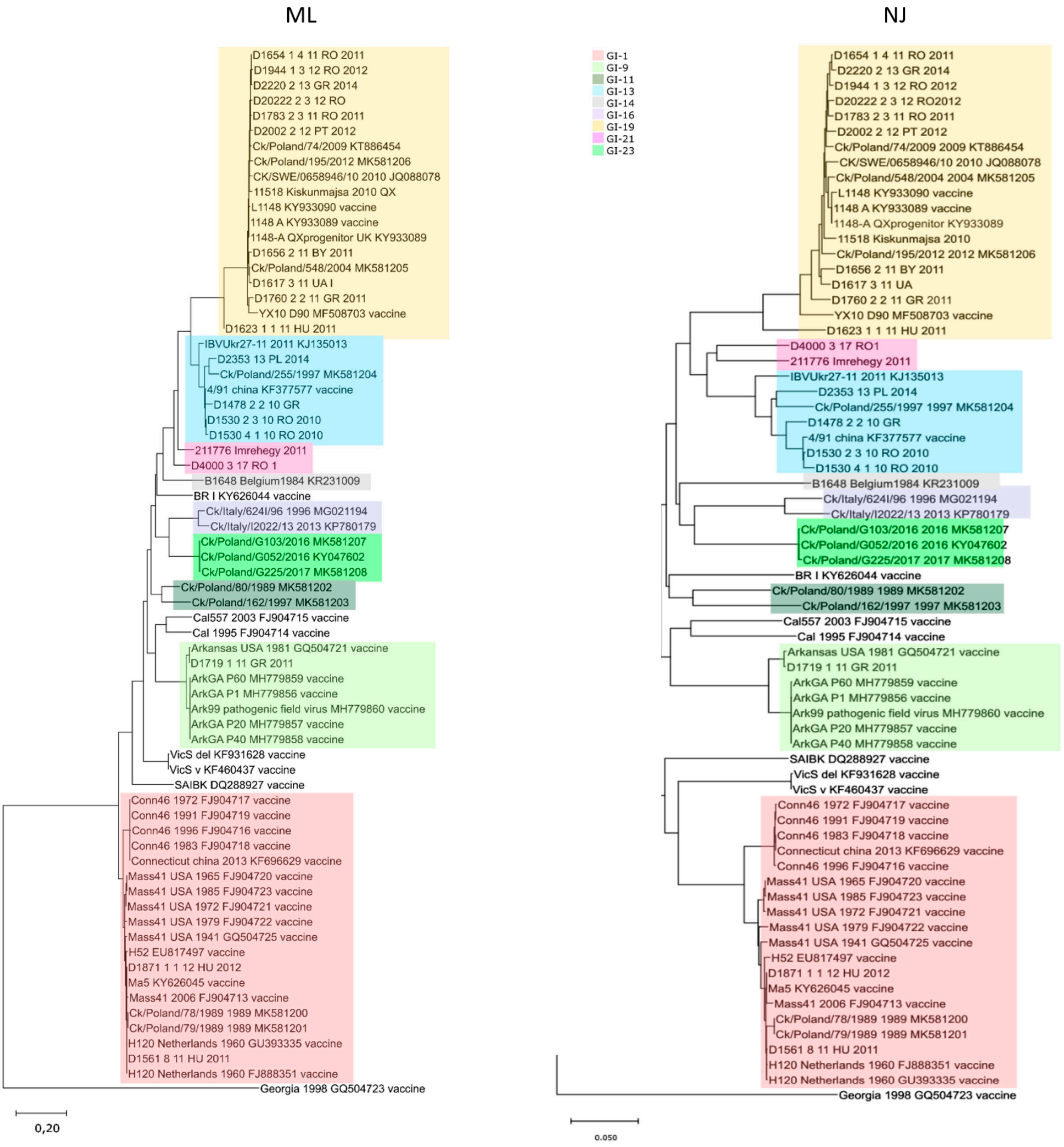

ML
NJ
GI-1
GI-9
GI-11
GI-13
GI-14
GI-16
GI-19
GI-21
GI-23
D1654 1 4 11 RO 2011
D1944 1 3 12 RO 2012
D2220 2 13 GR 2014
D20222 2 3 12 RO
D1783 2 3 11 RO 2011
D2002 2 12 PT 2012
Ck/Poland/74/2009 KT886454
Ck/Poland/195/2012 MK581206
CK/SWE/0658946/10 2010 JQ088078
11518 Kiskunmajsa 2010 QX
L1148 KY933090 vaccine
1148 A KY933089 vaccine
1148-A QXprogenitor UK KY933089
D1656 2 11 BY 2011
Ck/Poland/548/2004 MK581205
D1617 3 11 UA I
D1760 2 2 11 GR 2011
YX10 D90 MF508703 vaccine
D1623 1 1 11 HU 2011
IBVUkr27-11 2011 KJ135013
D2353 13 PL 2014
Ck/Poland/255/1997 MK581204
4/91 china KF377577 vaccine
D1478 2 2 10 GR
D1530 2 3 10 RO 2010
D1530 4 1 10 RO 2010
211776 Imrehegy 2011
D4000 3 17 RO 1
B1648 Belgium1984 KR231009
BR I KY626044 vaccine
Ck/Italy/6241/96 1996 MG021194
Ck/Italy/I2022/13 2013 KP780179
Ck/Poland/G103/2016 MK581207
Ck/Poland/G052/2016 KY047602
Ck/Poland/G225/2017 MK581208
Ck/Poland/80/1989 MK581202
Ck/Poland/162/1997 MK581203
Cal557 2003 FJ904715 vaccine
Cal 1995 FJ904714 vaccine
Arkansas USA 1981 GQ504721 vaccine
D1719 1 11 GR 2011
ArkGA P60 MH779859 vaccine
ArkGA P1 MH779856 vaccine
Ark99 pathogenic field virus MH779860 vaccine
ArkGA P20 MH779857 vaccine
ArkGA P40 MH779858 vaccine
VicS del KF931628 vaccine
VicS v KF460437 vaccine
SAIBK DQ288927 vaccine
Conn46 1972 FJ904717 vaccine
Conn46 1991 FJ904719 vaccine
Conn46 1996 FJ904716 vaccine
Conn46 1983 FJ904718 vaccine
Connecticut china 2013 KF696629 vaccine
Mass41 USA 1965 FJ904720 vaccine
Mass41 USA 1985 FJ904723 vaccine
Mass41 USA 1972 FJ904721 vaccine
Mass41 USA 1979 FJ904722 vaccine
Mass41 USA 1941 GQ504725 vaccine
H52 EU817497 vaccine
D1871 1 1 12 HU 2012
Ma5 KY626045 vaccine
Mass41 2006 FJ904713 vaccine
Ck/Poland/78/1989 1989 MK581200
Ck/Poland/79/1989 1989 MK581201
H120 Netherlands 1960 GU393335 vaccine
D1561 8 11 HU 2011
H120 Netherlands 1960 FJ888351 vaccine
Georgia 1998 GQ504723 vaccine
0,20
D1654 1 4 11 RO 2011
D2220 2 13 GR 2014
D1944 1 3 12 RO 2012
D20222 2 3 12 RO2012
D1783 2 3 11 RO 2011
D2002 2 12 PT 2012
Ck/Poland/74/2009 2009 KT886454
CK/SWE/0658946/10 2010 JQ088078
Ck/Poland/548/2004 2004 MK581205
L1148 KY933090 vaccine
1148 A KY933089 vaccine
1148-A QXprogenitor KY933089
11518 Kiskunmajsa 2010
Ck/Poland/195/2012 2012 MK581206
D1656 2 11 BY 2011
D1617 3 11 UA
D1760 2 2 11 GR 2011
YX10 D90 MF508703 vaccine
D1623 1 1 11 HU 2011
D4000 3 17 RO1
211776 Imrehegy 2011
IBVUkr27-11 2011 KJ135013
D2353 13 PL 2014
Ck/Poland/255/1997 1997 MK581204
D1478 2 2 10 GR
4/91 china KF377577 vaccine
D1530 2 3 10 RO 2010
D1530 4 1 10 RO 2010
B1648 Belgium1984 KR231009
Ck/Italy/6241/96 1996 MG021194
Ck/Italy/I2022/13 2013 KP780179
Ck/Poland/G103/2016 2016 MK581207
Ck/Poland/G052/2016 2016 KY047602
Ck/Poland/G225/2017 2017 MK581208
BR I KY626044 vaccine
Ck/Poland/80/1989 1989 MK581202
Ck/Poland/162/1997 1997 MK581203
Cal557 2003 FJ904715 vaccine
Cal 1995 FJ904714 vaccine
Arkansas USA 1981 GQ504721 vaccine
D1719 1 11 GR 2011
ArkGA P60 MH779859 vaccine
ArkGA P1 MH779856 vaccine
Ark99 pathogenic field virus MH779860 vaccine
ArkGA P20 MH779857 vaccine
ArkGA P40 MH779858 vaccine
SAIBK DQ288927 vaccine
VicS del KF931628 vaccine
VicS v KF460437 vaccine
Conn46 1972 FJ904717 vaccine
Conn46 1991 FJ904719 vaccine
Conn46 1983 FJ904718 vaccine
Connecticut china 2013 KF696629 vaccine
Conn46 1996 FJ904716 vaccine
Mass41 USA 1965 FJ904720 vaccine
Mass41 USA 1985 FJ904723 vaccine
Mass41 USA 1972 FJ904721 vaccine
Mass41 USA 1979 FJ904722 vaccine
Mass41 USA 1941 GQ504725 vaccine
H52 EU817497 vaccine
D1871 1 1 12 HU 2012
Ma5 KY626045 vaccine
Mass41 2006 FJ904713 vaccine
Ck/Poland/78/1989 1989 MK581200
Ck/Poland/79/1989 1989 MK581201
D1561 8 11 HU 2011
H120 Netherlands 1960 FJ888351 vaccine
H120 Netherlands 1960 GU393335 vaccine
Georgia 1998 GQ504723 vaccine
0.050

Whole genome

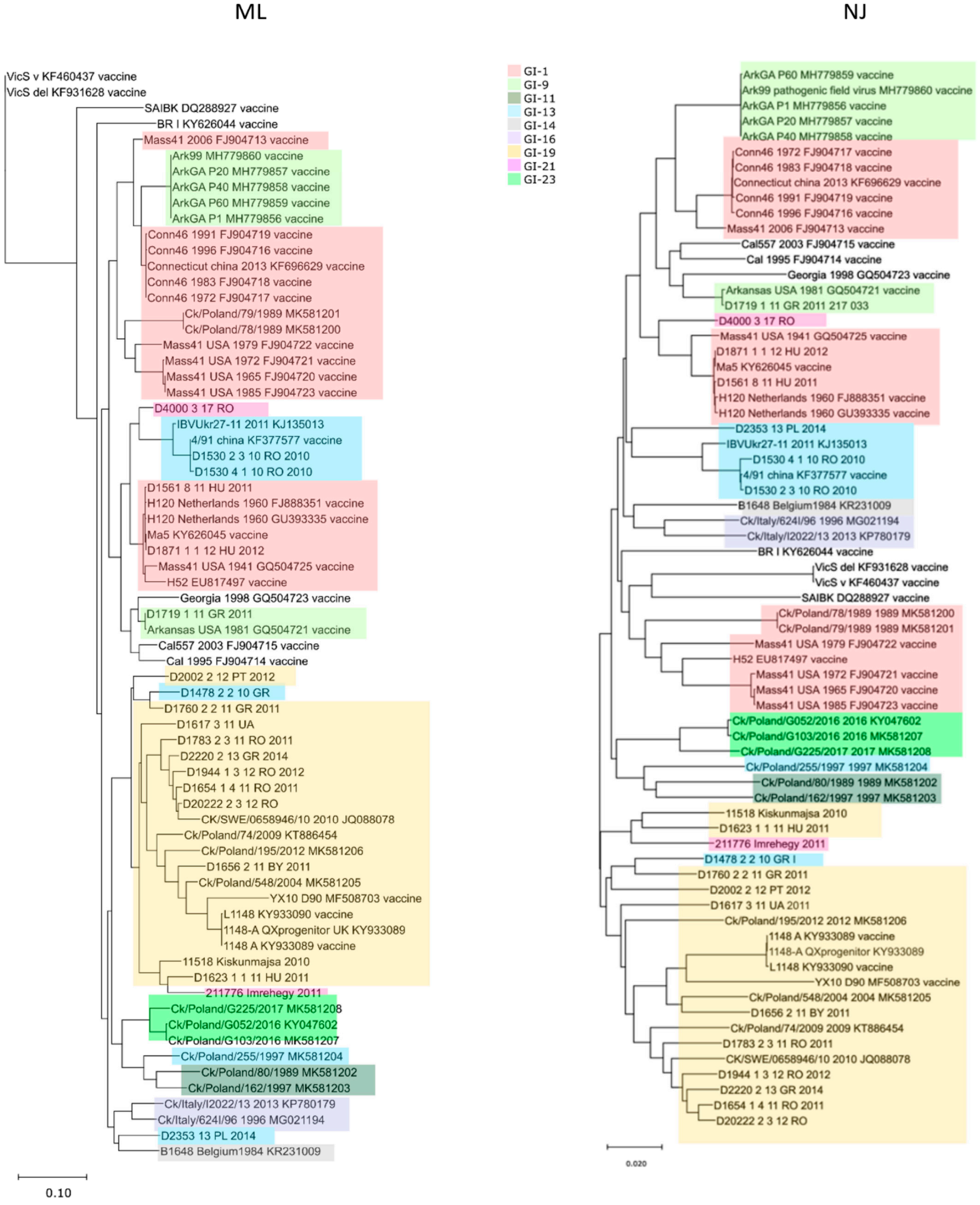

Supplement: Supplementary file 1 [file viruses-13-00535-s001.zip › Supplementary File S1.pdf]
