# Supplementary figures and images for: Recombination Events Shape the Genomic Evolution of Infectious Bronchitis Virus in Europe
Source: Viruses. 2021 Mar 24;13(4):535. doi: 10.3390/v13040535 (PMC8063831; doi:10.3390/v13040535)

# GI-1

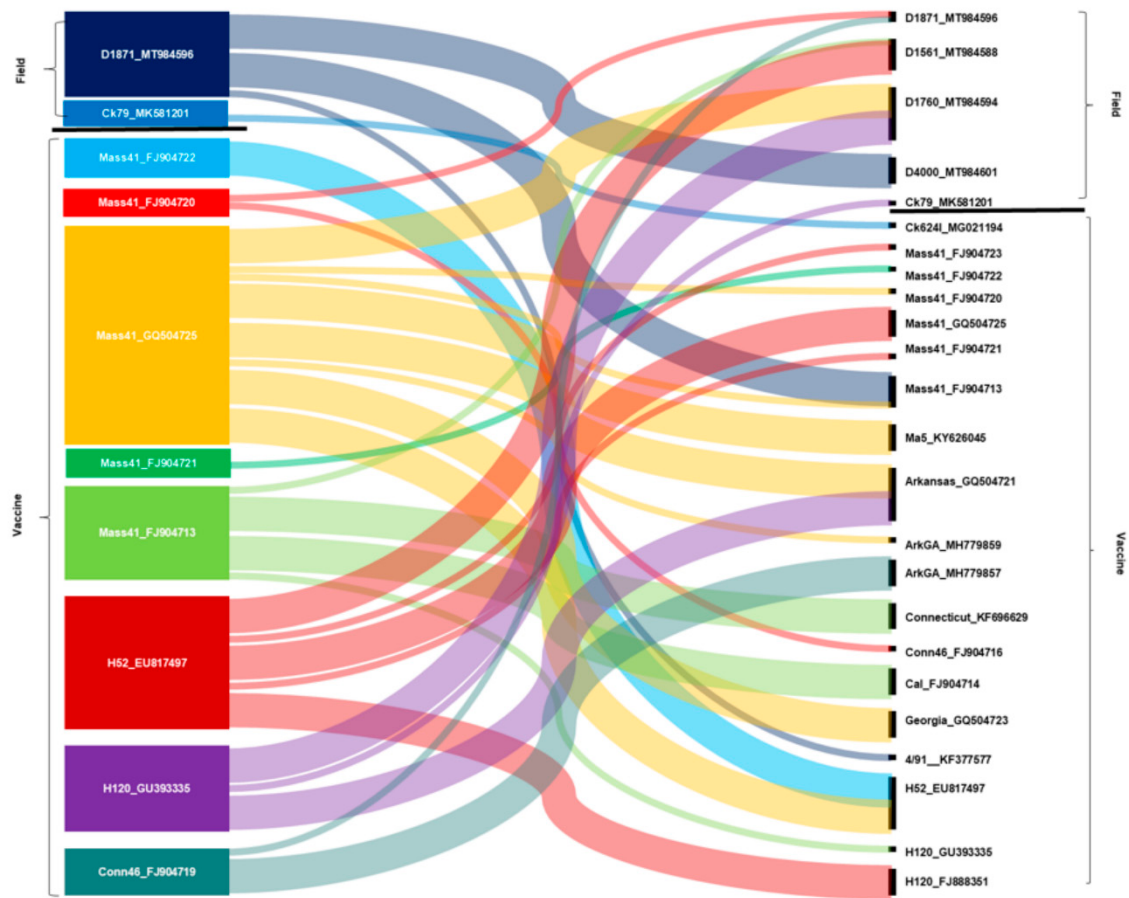

# GI-9

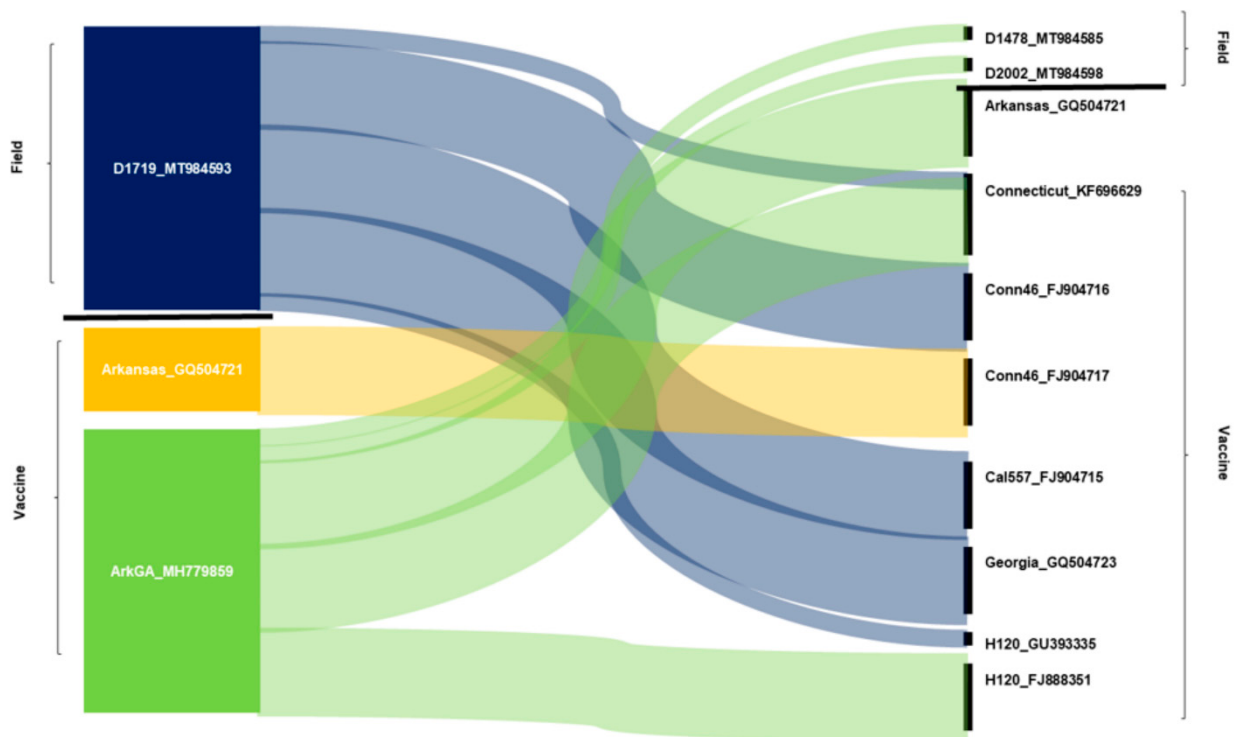

GI-11

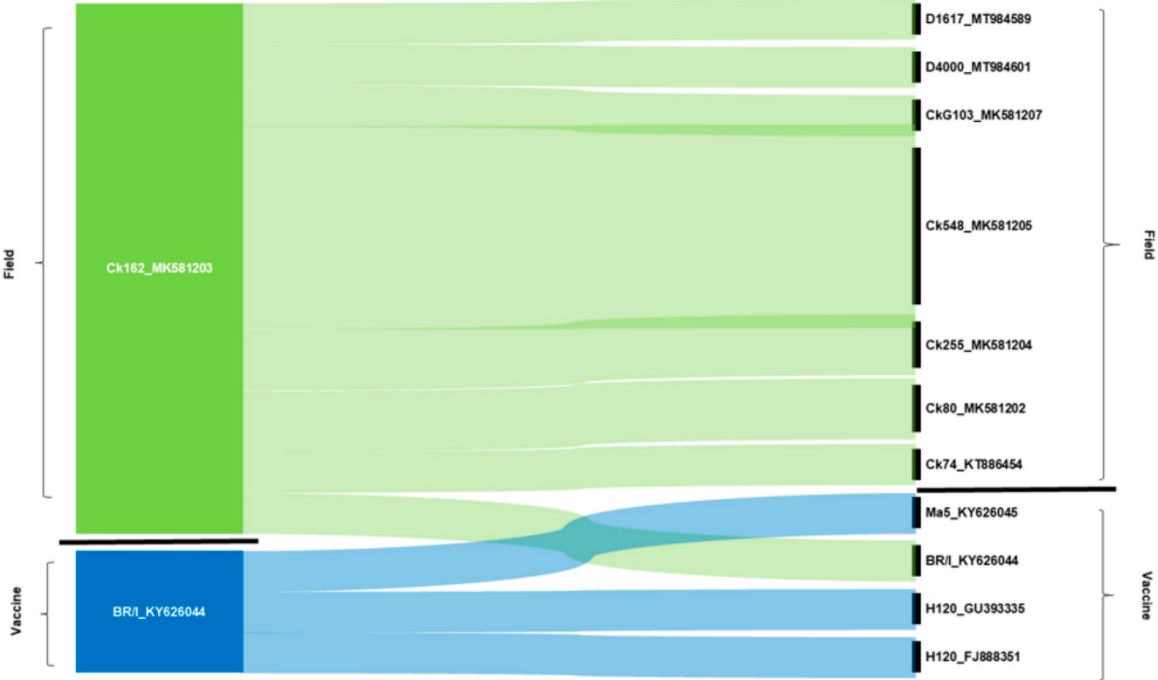

GI-13

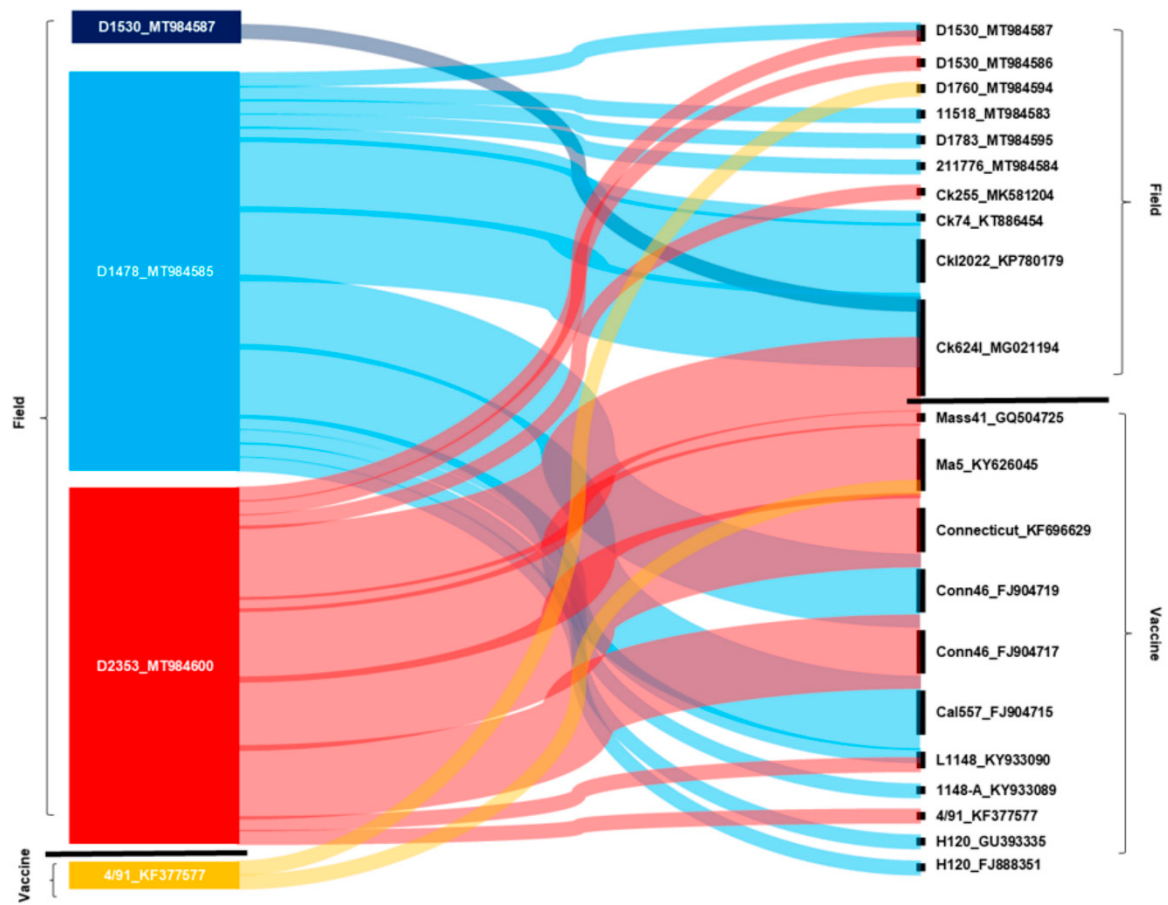

# GI-16

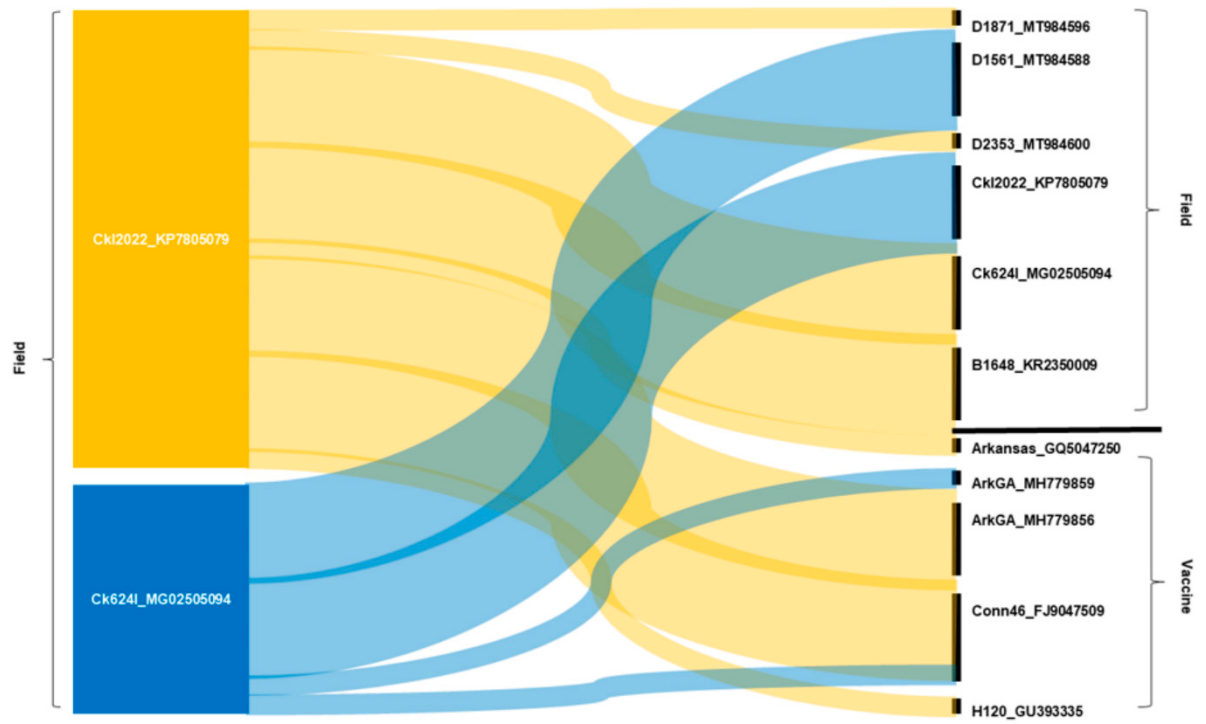

# GI-19

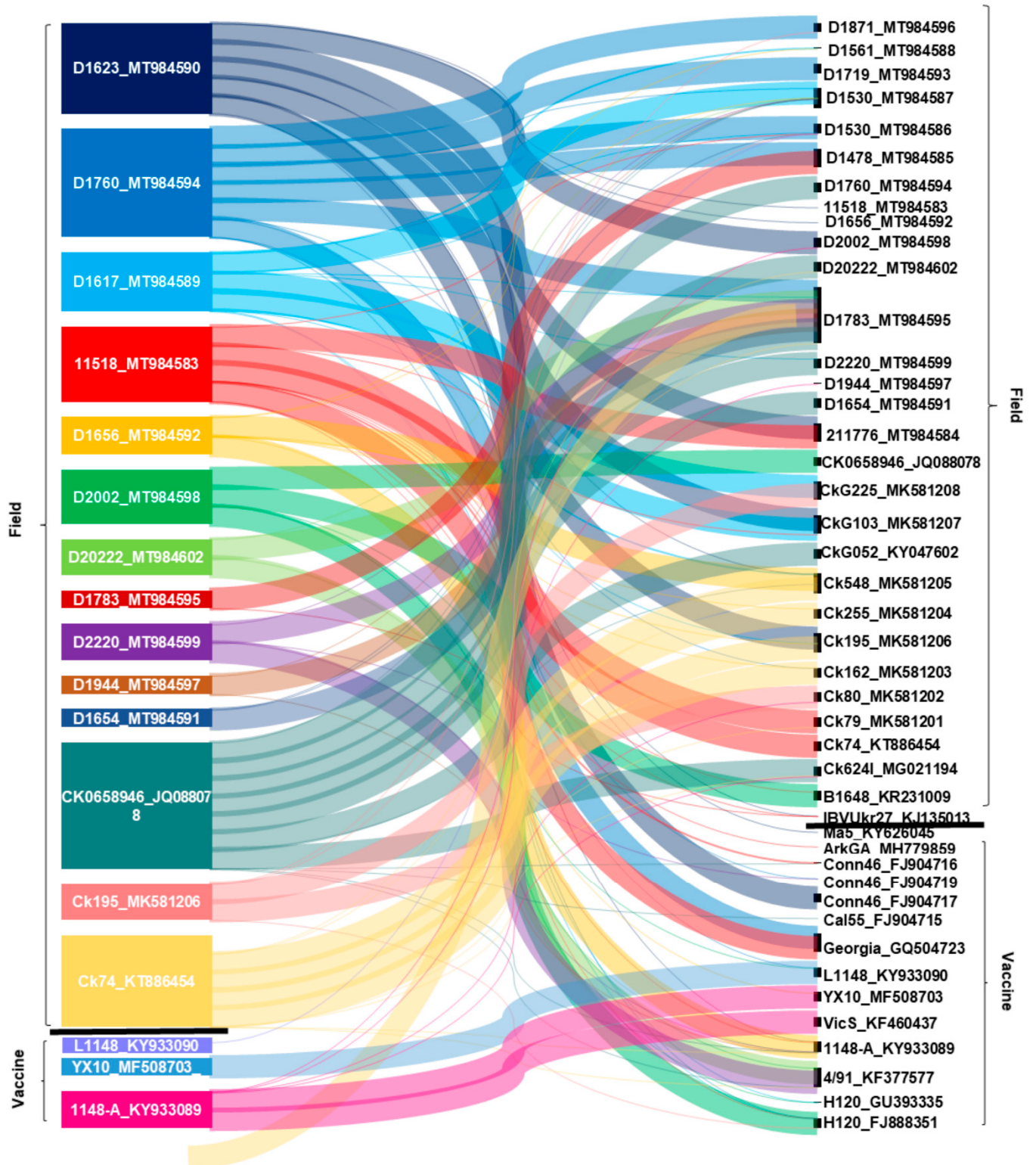

GI-21

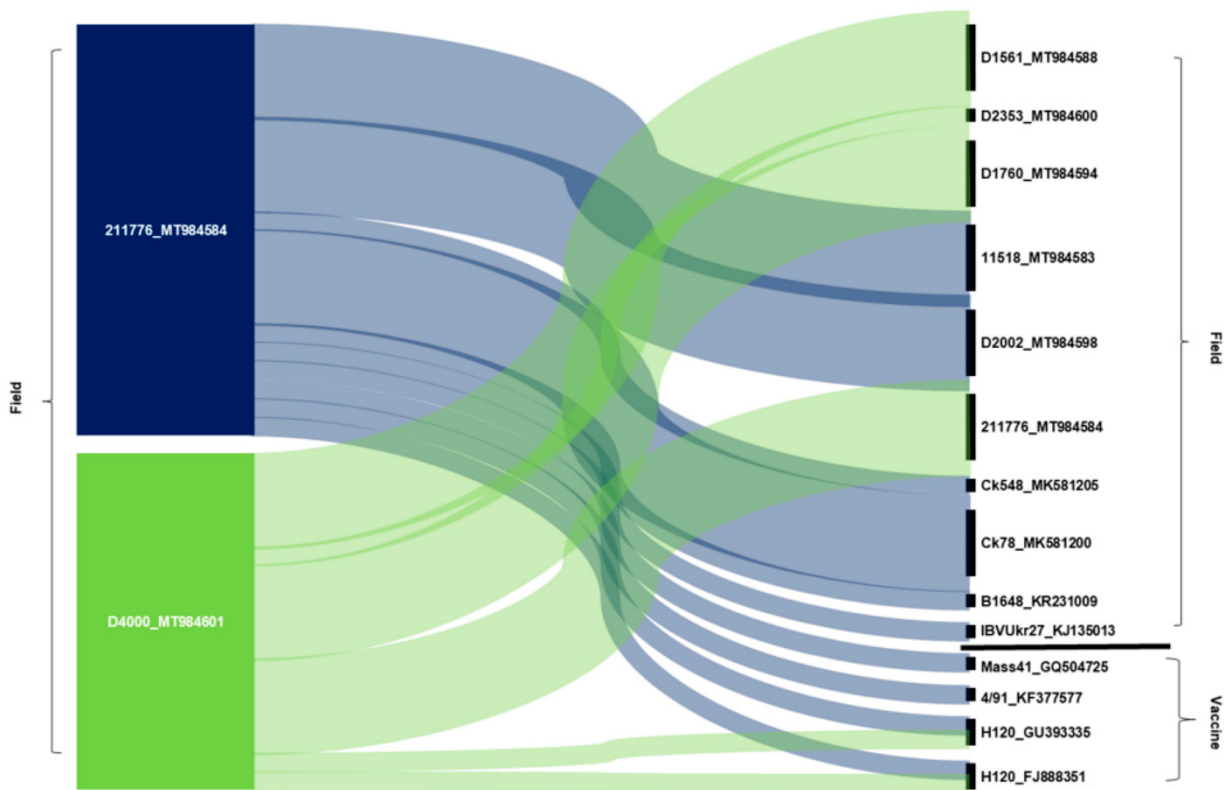

GI-23

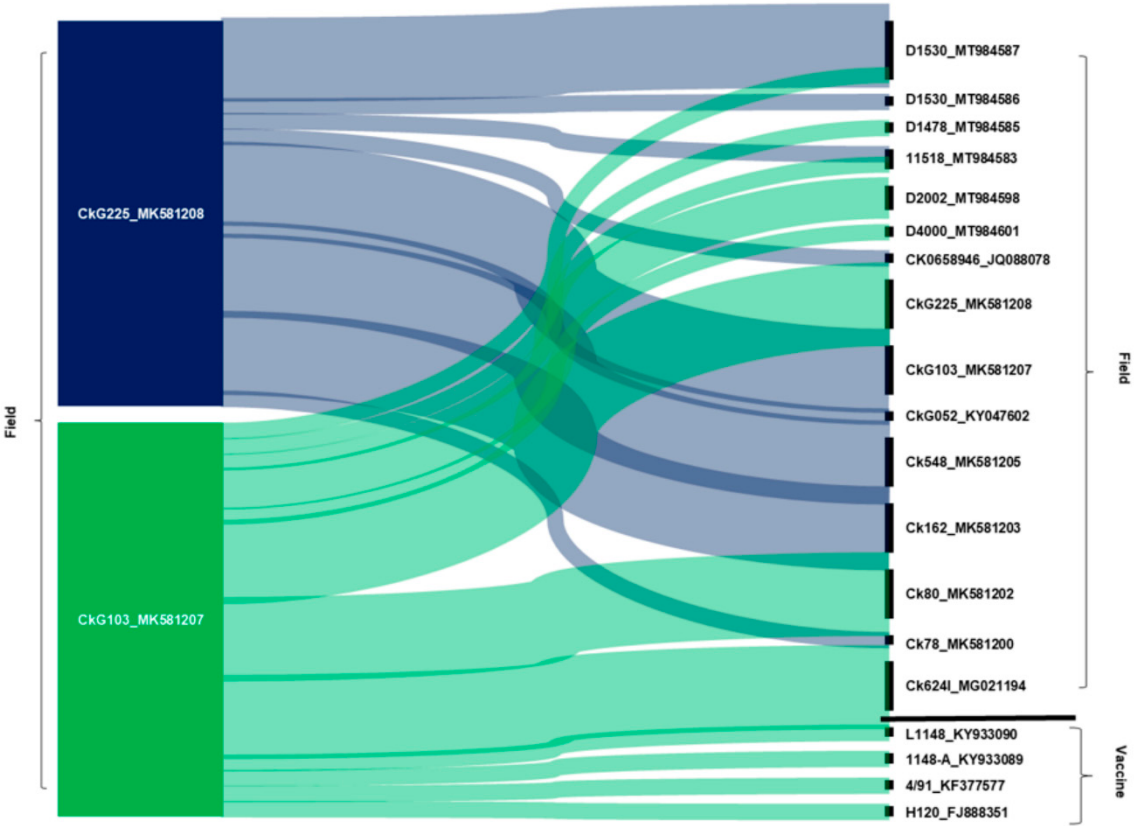

Supplement: Supplementary file 1 [file viruses-13-00535-s001.zip › Supplementary File S2.pdf]
